# GPIHBP1 on oligodendrocytes binds lipoprotein lipase within the human brain

**DOI:** 10.1073/pnas.2610646123

**Published:** 2026-06-01

**Authors:** Minjun Liu, Madison Hung, Ellen Kozlov, Megan Hung, Mariana Colaço-Gaspar, Shristi Roy, Yiping Tu, Shino D. Magaki, Christopher K. Williams, Maarja Andaloussi Mäe, Erik C. B. Johnson, Robert W. Siegel, Robert J. Konrad, Michael Ploug, Christer Betsholtz, Anne P. Beigneux, Liqun He, Loren G. Fong, Stephen G. Young

**Affiliations:** ^a^ Department of Medicine, David Geffen School of Medicine, University of California at Los Angeles, Los Angeles, CA 90095; ^b^ Department of Pathology and Laboratory Medicine, David Geffen School of Medicine, University of California at Los Angeles, Los Angeles, CA 90095; ^c^ Department of Immunology, Genetics, and Pathology, Rudbeck Laboratory, Uppsala University, Uppsala 75185, Sweden; ^d^ Department of Neurology, Emory University, Atlanta, GE 30329; ^e^Laboratory for Experimental Medicine, Lilly Research Laboratories, Eli Lilly and Company, Indianapolis, IN 46225; ^f^ Finsen Laboratory Copenhagen University Hospital–Rigshospitalet, Copenhagen N DK-2200, Denmark; ^g^ Biotechnology Research and Innovation Centre, University of Copenhagen, Copenhagen N DK-2200, Denmark; ^h^ Department of Medicine-Huddinge, Karolinska Institute Campus Flemingsberg, Huddinge SE-141 83, Sweden; ^i^ Department of Human Genetics, David Geffen School of Medicine, University of California at Los Angeles, Los Angeles, CA 90095

**Keywords:** GPIHBP1, lipoprotein lipase, oligodendrocyte, myelin

## Abstract

In peripheral tissues, lipoprotein lipase (LPL) is secreted by parenchymal cells (adipocytes, myocytes) into the interstitial spaces, where it is captured by GPIHBP1 (a glycosylphosphatidylinositol-anchored protein of capillary endothelial cells) and escorted to the luminal surface of capillaries. The LPL inside capillaries hydrolyzes glycerolipids in the plasma lipoproteins, releasing fatty acids for parenchymal cells. In the central nervous system, LPL is synthesized by multiple cell types [e.g., microglia, oligodendrocyte precursor cells (OPCs)] and secreted into the interstitium, but a binding site for the LPL has never been identified. By examining single nuclei RNA-seq databases of the human brain, we found that *GPIHBP1* is expressed by oligodendrocytes but not by OPCs. This gene-expression profile (high in oligodendrocytes, low in OPCs) is also observed in genes for myelin structural proteins, fatty acid binding and transport proteins, and lipid biosynthetic enzymes. *GPIHBP1* expression in oligodendrocytes was confirmed by in situ hybridization studies of human brain and by immunohistochemical staining. Of note, GPIHBP1 and LPL are colocalized on oligodendrocytes in the human brain. Our findings identify GPIHBP1 as a principal binding site for interstitial LPL in the human brain and suggest that GPIHBP1-bound LPL could hydrolyze interstitial lipids and thereby supply oligodendrocytes with fatty acid nutrients.

## Significance

In peripheral tissues, lipoprotein lipase (LPL) is synthesized by adipocytes and myocytes, secreted into the interstitial spaces, and then captured by GPIHBP1 on capillary endothelial cells. The LPL inside capillaries hydrolyzes triglycerides in lipoproteins and supplies myocytes and adipocytes with fatty acids. In the brain, LPL is synthesized by microglia (and other cell types) and secreted into the interstitium, but the binding site for the interstitial LPL has remained a mystery. Using single nuclei RNA-seq (snRNA-seq) analyses, in situ hybridization studies, and immunohistochemistry studies, we show that GPIHBP1 is expressed by oligodendrocytes and that it is a principal binding partner for LPL in the brain, positioning LPL to hydrolyze interstitial glycerolipids and supply oligodendrocytes with fatty acid nutrients.

The intravascular lipolytic processing of triglyceride (TG)-rich lipoproteins (TRLs) by lipoprotein lipase (LPL) provides fatty acids for storage in white adipose tissue and fuel for heart, skeletal muscle, and brown adipose tissue (1). In peripheral tissues, LPL is produced almost exclusively by parenchymal cells (adipocytes, myocytes), secreted into the interstitial spaces, and then captured by a glycosylphosphatidylinositol (GPI)-anchored protein (GPIHBP1) on the abluminal surface of capillary endothelial cells (ECs) (2–4). GPIHBP1 then transports LPL across ECs to the capillary lumen (2), where it hydrolyzes glycerolipids (mainly TGs but also phospholipids) in TRLs (5). In the absence of GPIHBP1, LPL is stranded within the interstitial spaces, preventing the intravascular lipolytic processing of TRLs and resulting in severe hypertriglyceridemia (2, 3).

GPIHBP1’s ability to bind LPL depends on two structural domains—a folded three-fingered LU (Ly6/uPAR) domain and an intrinsically disordered N-terminal acidic tail (6, 7). The LU domain interacts, largely by hydrophobic contacts, with LPL’s C-terminal lipid-binding domain (8, 9). *GPIHBP1* mutations that disrupt the conformation of the LU domain or perturb the GPIHBP1–LPL binding interface abolish LPL binding and transport and cause severe hypertriglyceridemia (10). GPIHBP1’s acidic domain, which contains many aspartates and glutamates as well as a sulfated tyrosine (11), interacts with a large basic patch on the surface of LPL (6, 9, 11). The acidic domain disrupts interactions between LPL and heparan sulfate proteoglycans (HSPGs) on the abluminal surface of capillaries and thereby frees LPL–GPIHBP1 complexes to move across ECs to the capillary lumen (12, 13). GPIHBP1’s acidic domain also increases the affinity of LPL binding and stabilizes the structure of LPL’s catalytic domain, thereby preserving LPL catalytic activity (14).

GPIHBP1 is not expressed by capillary ECs in the mouse brain parenchyma (15); consequently, intracapillary LPL and intravascular TRL processing are absent in mouse brain capillaries (5, 16, 17). In the mammalian brain, LPL is expressed in a variety of cell types [e.g., microglia, oligodendrocyte precursor cells (OPCs), neurons, fibroblasts, pericytes] and secreted into the interstitial spaces (18–25). The purpose of the interstitial LPL in the central nervous system (CNS) has never been defined, but it presumably functions to hydrolyze glycerolipids within the interstitial fluid (26). Although LPL expression in the CNS has been recognized and studied for more than 30 y (18–24), a binding site for LPL within the CNS has never been identified.

Most studies of LPL in the CNS have involved rodents (5, 16–19, 22), but we recently turned our attention to LPL and GPIHBP1 expression in the human CNS. In a recent study (27), we found *GPIHBP1* transcripts in ECs of the human choroid plexus (ChP) and *LPL* transcripts in human ChP pericytes and epithelial cells. Concurrently, we documented *Gpihbp1* transcripts in ECs of the mouse ChP, and we showed that the GPIHBP1 in mouse ChP capillaries binds LPL and transports it across ECs to the capillary lumen (27). The LPL inside ChP capillaries mediates both the margination and lipolytic processing of TRLs (27).

In the current study, we report that *GPIHBP1* is expressed by oligodendrocytes in the human brain. Oligodendrocytes produce the myelin that ensheathes nerve fibers and speeds transmission of nerve impulses. We hypothesized that oligodendrocyte GPIHBP1 functions to capture LPL within the interstitial fluid of the brain. In support of this idea, our studies revealed robust colocalization of GPIHBP1 and LPL on oligodendrocytes in the human brain.

## Results

### *GPIHBP1* and *LPL* Expression in the Human Brain.

**Fig. 1.**
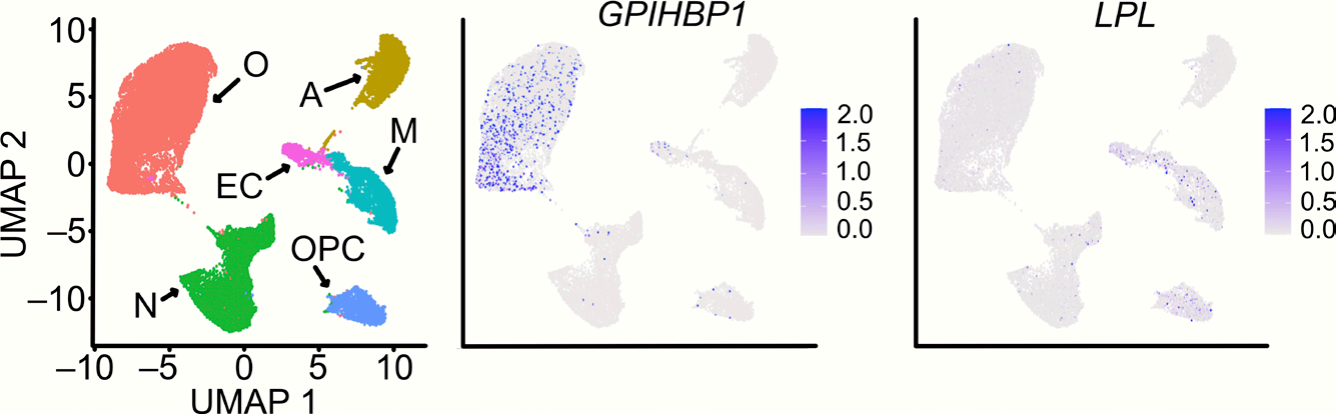
GPIHBP1 and LPL expression in human brain cell types. UMAP plots of single-nuclei RNA (snRNA-seq) data from a human brain (GSE253462) (30) showing expression of *GPIHBP1* and *LPL* across major cell types. *GPIHBP1* expression is enriched in oligodendrocytes (O) but is minimal in neurons (N), astrocytes (A), microglia (M), and oligodendrocyte precursor cells (OPCs). In contrast, *LPL* expression is highest in microglia, OPCs, and endothelial cells (ECs), with negligible expression in oligodendrocytes.

We found, by examining single nuclei RNA-seq (snRNA-seq) datasets of the human brain (28–30), that *GPIHBP1* is expressed in mature oligodendrocytes but not in oligodendrocyte precursor cells (OPCs), microglia, astrocytes, or neurons (Fig. 1). In an snRNA-seq study of the human cingulate cortex and substantia nigra (30), there were 1.15 *GPIHBP1* reads (per 10,000) in oligodendrocytes but fewer than 0.03 reads in astrocytes, neurons, and microglia. The level of *GPIHBP1* transcripts in oligodendrocytes was comparable to levels of *GPIHBP1* transcripts in ECs of the human heart (31). In the human heart, there were 0.473 *GPIHBP1* reads (per 10,000) in ECs but only 0.005 reads in cardiomyocytes and 0.009 reads in macrophages (31). *LPL* expression in the brain was detected, at low levels, in microglia, ECs, and OPCs, but *LPL* expression in oligodendrocytes was negligible (Fig. 1). In the human brain (30), there were 0.47 *LPL* reads (per 10,000) in microglia, 0.86 *LPL* reads in OPCs, and 0.06 *LPL* reads in neurons.

We examined *LPL* and *GPIHBP1* expression in the human hippocampus, cerebral cortex, heart, and skeletal muscle by qRT-PCR. *GPIHBP1* expression, relative to *GAPDH*, was higher in the cortex and hippocampus than in the heart (*SI Appendix,* Fig. S1), but those differences did not achieve statistical significance (*SI Appendix,* Fig. S1). *GPIHBP1* expression in the human hippocampus was higher than in skeletal muscle (*P* < 0.001) (*SI Appendix,* Fig. S1). *LPL* expression was higher in the human heart than in the human hippocampus, cerebral cortex, or skeletal muscle (*P* < 0.0001). The *GPIHBP1/LPL* expression ratio (from the *GAPDH*-normalized qRT-PCR data) was higher in the hippocampus and cerebral cortex than in the heart (*P* < 0.01) (*SI Appendix,* Fig. S1). When *GPIHBP1* and LPL expression data were normalized to *TUBB* (a commonly used “housekeeping” gene), *GPIHBP1* expression levels in the cortex, hippocampus, and heart were comparable to levels in the *GAPDH*-normalized data (*SI Appendix,* Fig. S2).

The qRT-PCR data showing comparable levels of *GPIHBP1* expression in the human brain and human heart were supported by data from the Genotype Tissue Expression (GTEx) project (32). *GPIHBP1* transcripts (per million) were 6.8 in the cerebral cortex, 14.9 in the hippocampus, 19.5 in the heart, and 13.3 in skeletal muscle. *LPL* transcripts (per million) were 8.6 in the cerebral cortex, 9.1 in the hippocampus, 234.4 in the heart, and 42.4 in the skeletal muscle.

Consistent with expression of *GPIHBP1* and *LPL* in the human brain, proteomic studies have identified GPIHBP1 and LPL in human cerebrospinal fluid (33–37).

**Fig. 2.**
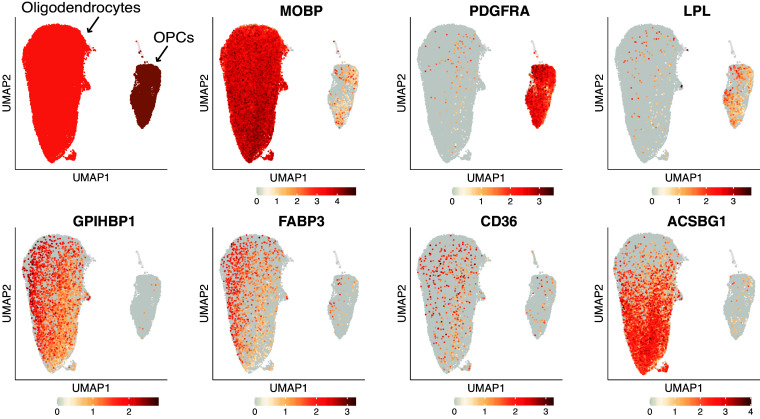
Gene-expression profiles in oligodendrocytes and oligodendrocyte precursor cells (OPCs). UMAP plots of human brain snRNA-seq data Siletti et al. (49) (https://github.com/linnarsson-lab/adult-human-brain, file: human_adult-GRCh38-3.0.0.h5ad) showing expression of *MOBP* (oligodendrocyte marker), *PDGFRA* (OPC marker), LPL, and genes for proteins involved in fatty acid metabolism and transport: *GPIHBP1*, *FABP3* (fatty acid binding protein 3), *CD36* (a plasma membrane fatty acid transporter), and *ACSBG1* (acyl-CoA synthetase bubblegum family member 1). *GPIHBP1* expression is enriched in oligodendrocytes, whereas *LPL* expression is higher in OPCs. 56,614 cells were analyzed.

**Fig. 3.**
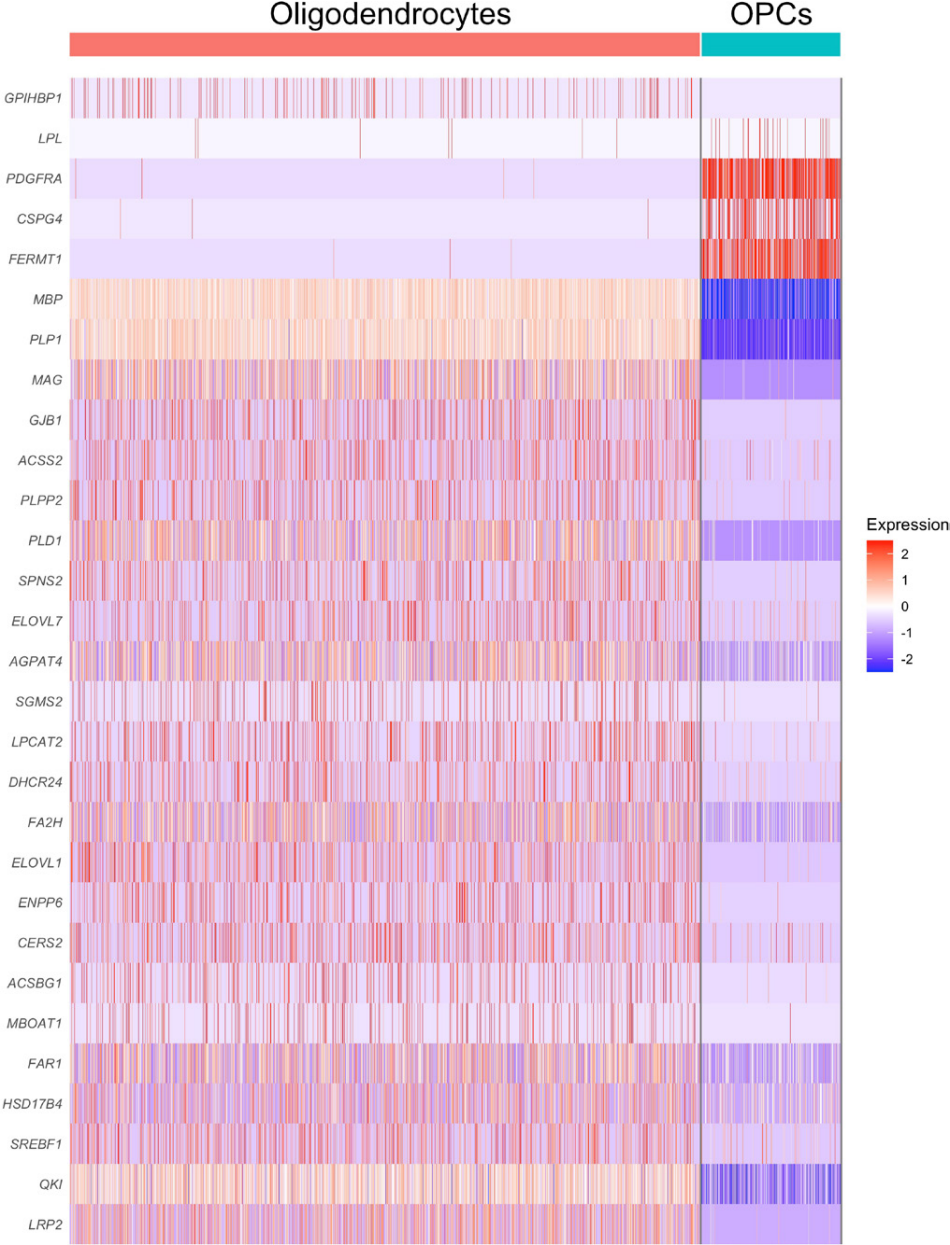
A heat map of gene expression in oligodendrocytes and OPCs. Heat map of snRNA-seq data from Siletti et al. (49) (https://github.com/linnarsson-lab/adult-human-brain, file: human_adult-GRCh38-3.0.0.h5ad) showing that the expression pattern for *GPIHBP1* (high in oligodendrocytes, low in OPCs) parallels that in genes for myelin structural proteins (MBP, PLP1, MAG, GJB1), lipid synthesis and metabolism proteins (ACSS2, PLPP2, PLD1, SPNS2, ELOV7, AGPAT4, SGMS2, LPCAT2, DHCR24, FA2H, ELOV1, ENPP6, CERS2, ACSBG1, MBOAT1, FAR1, HSD17B4), lipid regulatory factors (SREBF1, QKI), and LRP2 (a receptor that binds and internalizes APOE-containing lipoproteins). This pattern contrasts with *LPL* expression, which is higher in OPCs.

The pattern of *GPIHBP1* expression in the human brain (high in oligodendrocytes, low in OPCs) is shared by genes involved in fatty acid trafficking and metabolism (*FABP3, CD36, ACSBG1*) (Fig. 2). *LPL* exhibits the opposite pattern of expression (high in OPCs, low in oligodendrocytes) (Fig. 2). GPIHBP1’s expression pattern is shared by genes for myelin structural proteins (*MBP, PLP1, MAG, GJB1)*; genes related to lipid synthesis and metabolism (*ACSS2, PLPP2, PLD1, SPNS2, ELOV7, AGPAT4, SGMS2, LPCAT2, DHCR24, FA2H, ELOV1, ENPP6, CERS2, ACSBG1, MBOAT1, FAR1, HSD17B4*); genes that regulate lipid synthesis (*SREBF1, QKI*); and *LRP2*, which encodes a cell-surface receptor that binds and internalizes APOE-containing lipoproteins (Fig. 3). This same expression pattern is shared by genes associated with hypomyelination in humans or mice (*MBP, PLP1, ELOV1, ENPP6, CERS2, DHCR24*, *FA2H*, *FAR1*) (*SI Appendix,* Fig. S3) (38–48).

### *GPIHBP1* Expression in the CNS of Other Mammals.

**Fig. 4.**
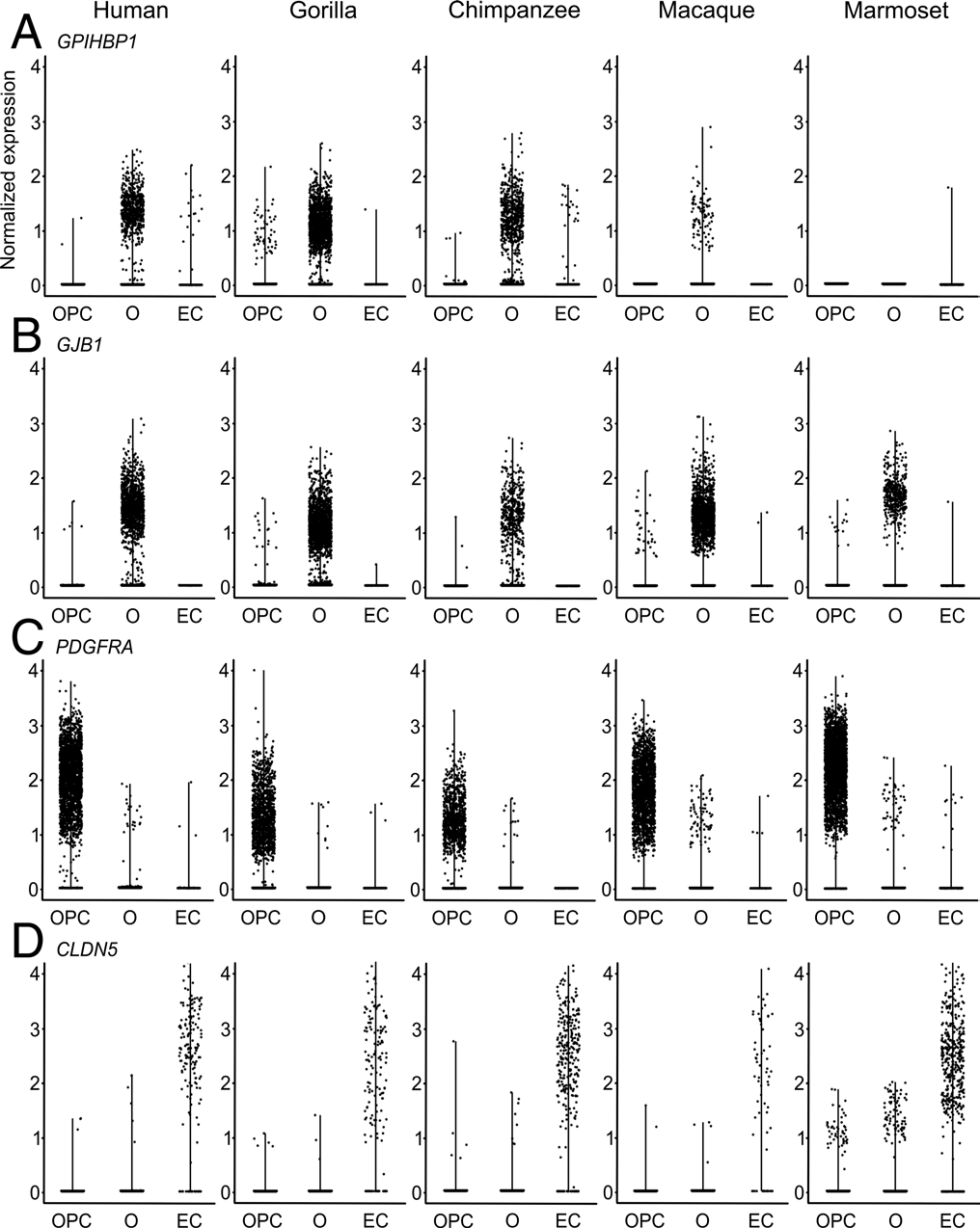
*GPIHBP1* expression in oligodendrocytes across primates. Single-nuclei RNA-seq data from Suresh et al. (51) showing normalized single-nuclei expression of *GPIHBP1* and cell type marker genes *GJB1* (oligodendrocytes, O), *PDGFRA* (oligodendrocyte precursor cells, OPC), and *CLDN5* (endothelial cells, EC) in five primates (human, gorilla, chimpanzee, macaque, marmoset). Each point represents an individual cell. *GPIHBP1* expression in oligodendrocytes is highest in large primates and absent in marmosets. Cell counts per species are as follows: human, 10,436; gorilla, 11,408; chimpanzee, 8,683; macaque, 11,599; marmoset, 7,246.

GPIHBP1 is not expressed by oligodendrocytes or OPCs in mice (50) (*SI Appendix,* Fig. S4), but it is expressed in oligodendrocytes of nonhuman primates, in particular large primates (51). Oligodendrocyte expression of *GPIHBP1* is robust in gorillas (~140 kg) and chimpanzees (~50 kg) and is found at lower levels in macaques (~9 kg) (Fig. 4). In marmosets (~0.5 kg), there is no *GPIHBP1* expression in oligodendrocytes. In human and chimpanzee brains, low levels of *GPIHBP1* transcripts were detected in ECs (Fig. 4).

### *GPIHBP1* Transcripts Are in Human Oligodendrocytes as Judged by In Situ Hybridization (ISH).

**Fig. 5.**
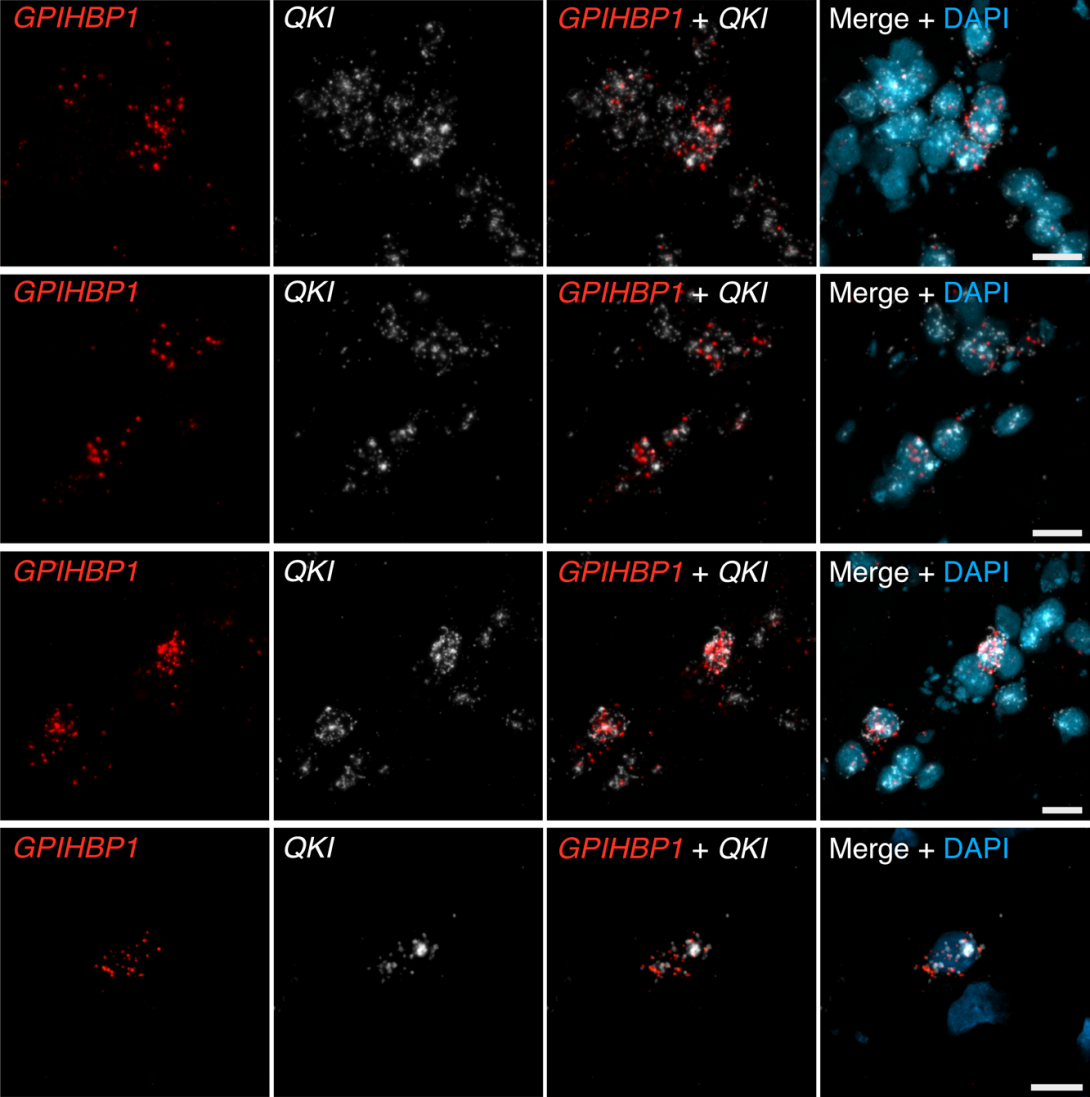
In situ hybridization studies with RNAscope probes showing colocalization of *GPIHBP1* and *QKI* transcripts in the human hippocampus. RNAscope probes were obtained from ACD Bio-Techne. In the first three rows, we used Dye 650 for *GPIHBP1* (*red*) and Dye 570 for *QKI* (*white*). In the last row, we used Dye 570 for *GPIHBP1* (*red*) and Dye 650 for *QKI* (*white*). Nuclei were stained with Dapi (*blue*). (Scale bar, 10 μm.)

ISH studies on postmortem human hippocampus revealed *GPIHBP1* transcripts in cells containing transcripts for *QKI* (Quaking) (Fig. 5). QKI is expressed at high levels in myelinating oligodendrocytes (Fig. 3) (52). As expected, transcripts for *GPIHBP1* and *PDGFRA* (an OPC marker) were observed in different cells (*SI Appendix,* Fig. S5*A*). *GPIHBP1* and *LPL* transcripts were also detected in different cells (*SI Appendix,* Fig. S5*B*). As expected, *GPIHBP1* transcripts could be detected in cells containing transcripts for OLIG2 (*SI Appendix,* Fig. S5*C*), a transcription factor that is expressed in both oligodendrocytes and OPCs (53). Some *OLIG2*-positive cells did not have *GPIHBP1* transcripts, consistent with *OLIG2* expression in OPCs.

### GPIHBP1 Is Expressed in Human Oligodendrocytes as Judged by Immunohistochemical Staining (IHC).

**Fig. 6.**
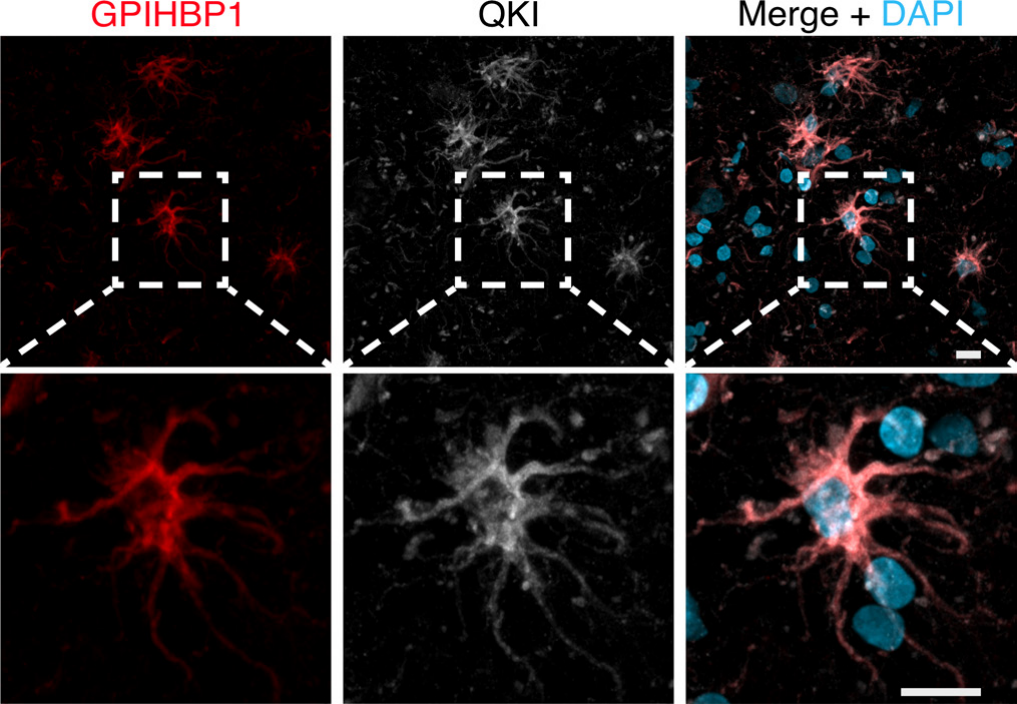
Colocalization of GPIHBP1 and QKI in human oligodendrocytes. Immunofluorescence staining of the human hippocampus showing GPIHBP1 and QKI in the same cells. GPIHBP1 was detected with an Alexa Fluor 647–labeled rabbit polyclonal antibody against human GPIHBP1 (*red*). QKI was detected with a mouse monoclonal antibody (CC1) followed by an Alexa Fluor 568–labeled donkey anti-mouse IgG (*white*). Nuclei were stained with Dapi (*blue*). (Scale bar, 10 μm.)

In preliminary studies, we assessed the binding of two independent QKI-specific monoclonal antibodies (mAbs) and two independent GPIHBP1-specific antibodies to formalin-fixed postmortem human hippocampus. Both QKI antibodies and both GPIHBP1 antibodies bound to cells with multiple branches and extensions [“ramified cells” (54)] (*SI Appendix,* Fig. S6). With those findings in hand, we tested whether the QKI and GPIHBP1 antibodies bound to the same cells. Those studies revealed robust colocalization of QKI and GPIHBP1 on ramified hippocampal cells (Fig. 6). We also observed colocalization of GPIHBP1- and OLIG2-specific antibodies on cells in the human frontal cortex, corpus callosum, and hippocampus (*SI Appendix,* Fig. S7). OLIG2 is a nuclear protein, but it escapes into the cytoplasm after death (55).

### LPL Colocalizes with GPIHBP1 on Oligodendrocytes of the Human Brain.

**Fig. 7.**
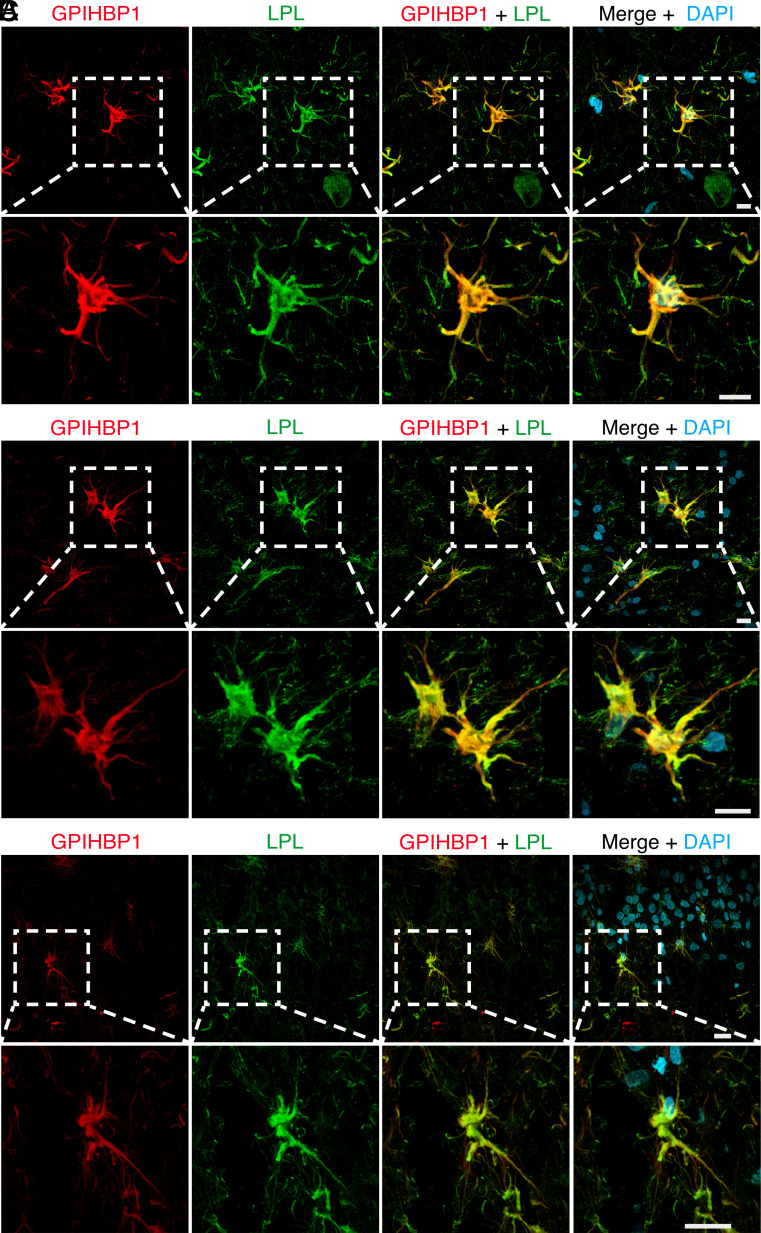
Colocalization of GPIHBP1 and LPL in the human hippocampus. Immunofluorescence staining showing colocalization of GPIHBP1 and LPL in ramified cells. Three fields are shown (*A*–*C*). GPIHBP1 was detected with a rabbit polyclonal antibody followed by an Alexa Fluor 568–labeled donkey anti-rabbit IgG (*red*). LPL was detected with a mouse monoclonal antibody (8G4) followed by an Alexa Fluor 647–labeled donkey anti-mouse IgG (*green*). Nuclei were stained with Dapi (*blue*). (Scale bars: 10 µm for *A* and *B*, 20 µm for *C*.)

**Fig. 8.**
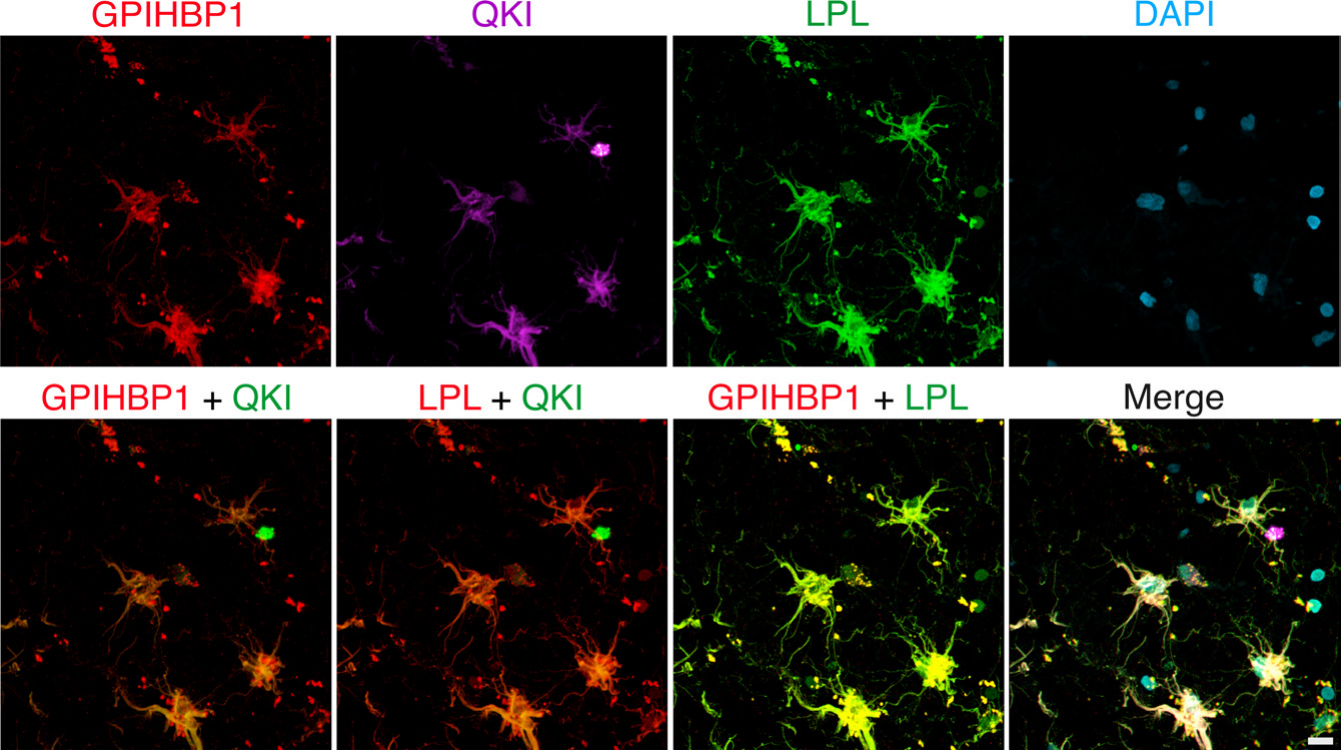
Colocalization of GPIHBP1, LPL, and QKI in human oligodendrocytes. Immunofluorescence staining of the human hippocampus. GPIHBP1 (Alexa Fluor 555–labeled rabbit IgG; *red*), QKI (mouse mAb CC1 followed by Alexa Fluor 647–labeled donkey anti-mouse IgG; *purple*), and LPL (Alexa Fluor 488–labeled rabbit IgG; *green*) colocalize on oligodendrocytes. Nuclei were stained with Dapi (*blue*). (Scale bar, 10 μm.)

In preliminary studies, we assessed binding of two independent human LPL–specific mouse mAbs to formalin-fixed postmortem human hippocampus. Both antibodies bound to ramified cells (*SI Appendix,* Fig. S8). We suspected that the GPIHBP1 on human oligodendrocytes represents a principal binding site for interstitial LPL in the human brain. Indeed, we observed robust colocalization of GPIHBP1 and LPL on the ramified cells in the human hippocampus (Fig. 7). Colocalization of LPL and GPIHBP1 was observed with two different human LPL–specific mAbs (*SI Appendix,* Fig. S9). We also observed robust colocalization of GPIHBP1, LPL, and QKI on oligodendrocytes in the human brain (Fig. 8 and *SI Appendix,* Fig. S10).

## Discussion

In peripheral tissues, the LPL that is secreted into the interstitium by adipocytes and myocytes is captured by a GPI-anchored protein, GPIHBP1, on the abluminal plasma membrane of capillary endothelial cells (2, 5). GPIHBP1 then escorts the LPL across ECs to the capillary lumen, where it hydrolyzes glycerolipids in TRLs and releases fatty acids for use by adipocytes and myocytes (56, 57). In the brain, several cell types (microglia, OPCs, neurons, pericytes) synthesize LPL and secrete it into the interstitial spaces (18–25). While LPL expression in the brain has been recognized for more than three decades (18–24), a binding site for LPL within the CNS has never been identified. Our current studies address that conundrum. By scrutinizing human brain snRNA-seq databases (28–30), we identified *GPIHBP1* expression in oligodendrocytes. *GPIHBP1* is also expressed in chimpanzee, gorilla, and macaque oligodendrocytes (51). Oligodendrocyte expression of *GPIHBP1* in the human brain was confirmed by in situ hybridization studies and by immunohistochemical staining. Remarkably, the levels of *GPIHBP1* transcripts in the human brain, as judged by qRT-PCR, are comparable to levels in the human heart (an organ where GPIHBP1 is important for the intravascular processing of TRLs) (56). The ratio of *GPIHBP1* to *LPL* expression is greater in the human hippocampus and cerebral cortex than in the human heart.

We suspected that the GPIHBP1 on oligodendrocytes functions to capture LPL from the interstitium of the brain—analogous to GPIHBP1’s function in ECs of peripheral tissues (2, 4). Consistent with this suspicion, our immunohistochemistry studies revealed robust colocalization of GPIHBP1 and LPL on ramified cells that express oligodendrocyte marker proteins (QKI, OLIG2). The binding of LPL by GPIHBP1 is likely important because biophysical studies have revealed that GPIHBP1 stabilizes the conformation of LPL’s hydrolase domain and preserves LPL catalytic activity (14, 58–61).

*GPIHBP1* is expressed by human oligodendrocytes but not OPCs; this gene-expression pattern is shared by genes for a variety of myelin structural proteins and lipid biosynthetic enzymes. This observation alone raised the possibility that GPIHBP1 expression in oligodendrocytes is related to the principal function of oligodendrocytes, which is to produce myelin. We suspect that GPIHBP1-bound LPL on oligodendrocytes hydrolyzes glycerolipids in interstitial lipoproteins (62) and cellular debris [including the myelinoid bodies released by oligodendrocytes (63)], thereby supplying oligodendrocytes with fatty acid nutrients. The genes for CD36 (a plasma membrane fatty acid transporter), FABP3 (a cytosolic fatty acid binding protein), and ACSBG1 (an enzyme that converts long-chain fatty acids into acyl-CoA thioesters) are expressed in mature oligodendrocytes but not OPCs (mirroring *GPIHBP1*’s expression profile).

LPL, when bound to GPIHBP1, is catalytically active (11, 14, 58, 61). For that reason, it makes sense that GPIHBP1–LPL complexes on the oligodendrocyte plasma membrane would be capable of hydrolyzing interstitial lipids in the CNS. Earlier studies by Yagyu et al. (64) provide strong support for this proposal. They created transgenic mice expressing a GPI-anchored LPL in cardiomyocytes. As expected, the LPL in those mice was anchored to the plasma membrane of cardiomyocytes and was not transported into capillaries. The GPI-anchored LPL triggered lipid accumulation in cardiomyocytes, leading the authors to conclude that it was active in hydrolyzing interstitial glycerolipids and thereby promoting intracellular lipid accumulation (64). Similarly, we suspect that LPL–GPIHBP1 complexes on oligodendrocytes hydrolyze interstitial lipids and promote lipid uptake into oligodendrocytes. An activator of LPL catalytic activity, APOC2, is present within the CNS (65).

In peripheral cells, LPL’s principal biochemical function is to hydrolyze TGs in TRLs, but the lipoproteins in brain interstitial fluid are phospholipid-rich and have very low levels of TGs (66). Of note, LPL has been reported to have substantial phospholipase activity (67, 68), and our own experiments have been consistent with those findings (*SI Appendix,* Fig. S11). Since LPL can hydrolyze phospholipids, the notion that LPL in the brain hydrolyzes phospholipids in interstitial lipoproteins and cellular debris is reasonable. Astrocytes, the predominant source of lipoproteins in the brain, are intimately associated with oligodendrocytes in the brain parenchyma (69).

A MRI study of marathon runners revealed that the myelin content of motor-coordination regions of the brain is reduced after a marathon but recovers within 2 mo, implying that myelin serves as an energy reserve during extreme exercise (70). We suspect that GPIHBP1-bound LPL on oligodendrocytes facilitates clearance of oligodendrocyte debris during extreme exercise and promotes the recovery of myelin lipid stores by releasing fatty acids from interstitial lipoproteins in the brain.

Several members of the pancreatic lipase family (LPL, endothelial lipase, and hepatic lipase) have been proposed to exhibit transacylase activity (the ability to transfer an acyl group from a lipid donor to an acceptor lipid) (71). With each of these enzymes, hydrolysis is the dominant reaction in an aqueous environment; however, transacylation can occur when lipid acceptors are abundant and therefore able to outcompete water in the enzymatic reaction (72). It is conceivable that LPL and lipid acceptors are abundant in the “oligodendrocyte microenvironment.” Transacylation of lysophosphatidylcholine (lyso-PC) to generate phosphatidylcholine (PC) could limit accumulation of lyso-PC, which has been shown to cause demyelination in peripheral nerves (73).

The lipolytic processing of interstitial lipoproteins by LPL is probably not the sole mechanism by which oligodendrocytes obtain lipids for myelin production. LRP2, a cell-surface receptor that binds and internalizes astrocyte-derived APOE-containing lipoproteins (74), is expressed by oligodendrocytes but not OPCs. Astrocyte-derived lipoproteins are important for both myelin production in oligodendrocytes and oligodendrocyte viability (75, 76).

While it makes sense that GPIHBP1-bound LPL would be useful for supplying oligodendrocytes with fatty acids, we do not believe that this mechanism is crucial in humans. Deficiencies of *GPIHBP1* and *LPL* cause severe hypertriglyceridemia, but overt neurological deficits have not been described. The absence of neurological disease is not particularly surprising. In the heart, LPL synthesis and intravascular LPL-mediated TRL processing is robust, but cardiac pathology has not been reported in humans with *GPIHBP1* or *LPL* deficiency, likely because cardiomyocytes are able to use glucose and plasma free fatty acids for fuel. In the absence of *GPIHBP1* or LPL, we suspect that de novo lipogenesis and LRP2-mediated lipoprotein uptake in oligodendrocytes are sufficient to prevent overt neuropathology.

Oligodendrocyte expression of GPIHBP1 is robust in large primates (human, gorilla, chimpanzee), modest in a smaller primate (macaque), and absent in a tiny primate (marmoset) (51). GPIHBP1 expression is also absent in oligodendrocytes in the mouse brain. These differences in GPIHBP1 expression likely reflect differences in brain size and complexity. White matter makes up to 40 to 50% of the human brain but only ~10 to 15% of mouse and marmoset brains (77–81). Given the reduced requirement for myelin production in small mammals, we suspect that lipoprotein uptake and de novo lipogenesis are sufficient for myelin production.

Aside from the differences in oligodendrocyte *GPIHBP1* expression in humans and mice, there is discrepant expression of *GPIHBP1* in ECs. GPIHBP1 is absent from blood–brain barrier (BBB) ECs in mice (15), but snRNA-seq studies of the human brain have uncovered *GPIHBP1* expression in ECs (28–30, 49). Of note, however, *GPIHBP1* expression in humans and chimpanzee brains was greater in oligodendrocytes than in ECs, as judged by snRNA-seq studies (51). In our ISH and IHC experiments on the postmortem human brain, we observed robust expression of GPIHBP1 in oligodendrocytes but did not find convincing examples of GPIHBP1 expression in capillary ECs.

GPIHBP1 and LPL physiology in peripheral tissues and the brain are very distinct, but there is a commonality. In both settings, the cells that make LPL and GPIHBP1 are different. In peripheral tissues, LPL is produced predominantly by adipocytes and myocytes, whereas GPIHBP1 is produced by capillary ECs. In the brain parenchyma, LPL is produced predominantly by microglia, OPCs, neurons, and pericytes, whereas GPIHBP1 is expressed in oligodendrocytes (28–30, 49). Most of the LPL in heart tissue sections is on capillary ECs rather than on cardiomyocytes (which synthesize nearly all the LPL) (2, 4). Similarly, most of the LPL protein in sections of the human brain is on GPIHBP1-expressing oligodendrocytes rather than on the cells producing the LPL. At this point, we do not fully understand why nature has separated sites of LPL and GPIHBP1 synthesis, but we suspect that this arrangement relates to the capacity of GPIHBP1 to stabilize the conformation of LPL’s hydrolase domain (11, 14, 59, 61, 82). By stabilizing LPL conformation, GPIHBP1 prevents unfolding of LPL at body temperature, thereby preserving LPL catalytic activity (58). Thus, LPL catalytic activity is expected to be higher at sites where GPIHBP1 is present—capillary endothelial in peripheral tissues and the surface of oligodendrocytes in the brain.

## Materials and Methods

### Human Tissues.

Deidentified frozen human tissue was obtained from UCLA’s Tissue Procurement Core and the Neuropathology Core at the Mary S. Easton Center for Alzheimer’s Research and Care (operating under UCLA IRB approval 11-2504). UCLA neuropathologists harvested brain tissue within 24 h after death.

### Antibodies.

Human LPL was detected with mouse monoclonal antibodies 8G4 and 88B8 (83) and a protein A–purified rabbit polyclonal antibody against recombinant human LPL (27). GPIHBP1 was detected with an immunopurified rabbit polyclonal antibody against recombinant human GPIHBP1 and by a cocktail of three human GPIHBP1 monoclonal antibodies (RE3, RG3, RF4) (84). QKI was detected with mouse monoclonal antibodies CC1 (Abcam, ab16794) (85) and N147/6 (Abcam, ab186245). OLIG2 was detected with mouse monoclonal antibody 211F1.1 (Sigma-Aldrich, 387 M-1). Alexa Fluor dye–labeled secondary antibodies were purchased from Thermo Fisher Scientific and Jackson ImmunoResearch.

### Immunohistochemical Studies.

10-μm-thick frozen sections of tissue samples were prepared, fixed with methanol or paraformaldehyde for 10 min, and incubated in blocking buffer (PBS containing 0.2% BSA and 5% donkey serum) for 1 h at room temperature (27). Sections were incubated with primary antibodies at 4 °C overnight. Sections were washed three times to remove unbound antibodies and then incubated with Alexa Fluor–labeled secondary antibodies for 45 min. After washing, the sections were postfixed with 3% PFA for 5 min, and cell nuclei were stained with DAPI. Images were recorded on LSM800 or LSM980 microscopes (Zeiss) with 20× or 63× objectives (27).

### In Situ Hybridization (ISH) Studies.

ISH experiments were performed with RNAscope probes and the RNAscope Multiplex Fluorescent Detection Kit v2.0 (ACD Bio Techne, CA, USA) according to procedures described previously (27). Paired double-Z oligonucleotide probes for *GPIHBP1, LPL, QKI, OLIG2,* and *PDGFRA* were purchased from ACD Bio Techne (GPIHBP1, 1811681-C1; QKI, 416351-C3; OLIG2, 424191-C3; LPL 530851-C2; PDGFRA, 604481-C2). 10-µm-thick cryosections were incubated in 3% PFA for 1 h at 4 °C, dehydrated through ethanol series (50, 70, and 100%), and then rehydrated in distilled water before inactivating endogenous peroxidase with H_2_O_2_ for 10 min at room temperature. PretreatPro Reagent was then applied to the sections for 30 min. RNAscope probes were hybridized for 2 h at 40 °C in a HybEZ II Oven. Probe signals were sequentially amplified with AMP 1 to 3 and then developed with tyramide signal amplification (TSA) with Opal 570 and Opal 650 dyes. Sections were counterstained with DAPI before mounting. Images were recorded on an LSM980 microscope equipped with a 63× objective (Objective Plan-Apochromat 63×/1.4 Oil DIC M27 lens from Zeiss with 353/465-nm, 493/517-nm, 548/561-nm, and 650/673-nm excitation/emission filters) (27).

### Single-Cell and Single-Nuclei Transcriptomics.

To characterize the expression patterns of GPIHBP1 and LPL in the human brain, we analyzed publicly available snRNA-seq datasets (GSE253462, GSE118257, GSE175814) (28–30, 49). For quality control, we excluded cells with >6% mitochondrial gene content, those expressing fewer than 1,000 or more than 6,000 genes, and cells with unique molecular identifier (UMI) counts exceeding 30,000. A single-cell transcriptomic dataset for mouse brain oligodendrocytes was obtained from a previously published study from (50) (NCBI GEO accession: GSE75330), comprising a total of 5,069 cells. The expression of *GPIHBP1* in oligodendrocytes and oligodendrocyte precursor cells were retrieved from the adult brain snRNA-seq study (49) with raw files downloaded from GitHub repository (https://github.com/linnarsson-lab/adult-human-brain; file: human_adult-GRCh38-3.0.0.h5ad). To reduce computational complexity and facilitate comparative analyses, a random 10% subsample of the original dataset was used, resulting in 56,614 cells included in downstream analysis. For cross-species comparisons among five primates (human, chimpanzee, gorilla, macaque, and marmoset), data were obtained from a published dataset (51) and downloaded through the Neuroscience Multi-omics Archive (NeMO ID: dat-net1412).

Transcriptomic analyses were performed according to procedures that we have described previously (27). Briefly, raw data were processed using Seurat (v5.0.3) in R (v4.3.0). Raw counts were normalized using a scale factor of 10,000 reads per cell, and variable genes were identified using the variance-stabilizing transformation (vst) method. The resulting data matrices were scaled and subjected to principal component analysis (PCA) for dimensionality reduction. A shared nearest neighbor (SNN) graph was constructed using the FindNeighbors function, and Uniform Manifold Approximation and Projection (UMAP) was used for visualization and identification of cell populations.

### qRT-PCR Studies.

Human tissues (cerebral cortex, hippocampus, heart, and skeletal muscle) were snap-frozen and placed into TRIzol buffer (Thermo Fisher Scientific) for RNA isolation (27). Total RNA was extracted with the RNeasy kit (Qiagen) and treated with DNase I (Ambion) (27). RNA was reverse-transcribed with random primers and the SuperScript III cDNA Synthesis Kit (Invitrogen). qRT-PCR reactions were performed on a QuantStudio5 system (Thermo Fisher Scientific) with SYBR Green PCR Master Mix (Bioland). Transcript levels were calculated by the comparative cycle threshold method (27). Oligonucleotide primers are listed in the *SI Appendix*, Table S1).

## Supplementary Material

Appendix 01 (PDF)

## Data Availability

Study data are included in the article and/or *SI Appendix*.
