# Supplementary material for: GPIHBP1 on oligodendrocytes binds lipoprotein lipase within the human brain
Source: Proc Natl Acad Sci U S A. 2026 Jun 1;123(23):e2610646123. doi: 10.1073/pnas.2610646123 (PMC13250511; doi:10.1073/pnas.2610646123)
Supplement: Supplementary file 1 — Appendix 01 (PDF) [file pnas.2610646123.sapp.pdf]

PNAS
www.pnas.org

Supplementary Information for:

GPIHBP1 on oligodendrocytes binds lipoprotein lipase within the human brain

Minjun Liu[a,*], Madison Hung[a,*], Ellen Kozlov[a,*], Megan Hung[a], Mariana Colaço-Gaspar[a], Shristi Roy[a], Yiping Tu[a], Shino D. Magaki[b], Christopher K. Williams[b], Maarja Andaloussi Mäe[c], Erik C. B. Johnson[d], Robert W. Siegel[e], Robert J. Konrad[e], Michael Ploug[f,g], Christer Betsholtz[c,h], Anne P. Beigneux[a], Liqun He[c,†], Loren G. Fong[a,†], and Stephen G. Young[a,i,†]

[a]Department of Medicine, David Geffen School of Medicine, UCLA, Los Angeles, CA, USA; [b]Department of Pathology and Laboratory Medicine, David Geffen School of Medicine, UCLA, Los Angeles, CA, USA; [c]Department of Immunology, Genetics, and Pathology, Rudbeck Laboratory, Uppsala University, Uppsala, Sweden; [d]Department of Neurology, Emory University, Atlanta, GE; [e]Lilly Research Laboratories, Eli Lilly and Company, Indianapolis, Indiana 46225 USA; [f]Finsen Laboratory, Copenhagen University Hospital - Rigshospitalet, DK–2200 Copenhagen N, Denmark; [g]Biotechnology Research and Innovation Centre (BRIC), University of Copenhagen, DK-2200 Copenhagen N, Denmark; [h]Department of Medicine-Huddinge, Karolinska Institute Campus Flemingsberg, Huddinge, Sweden; [i]Department of Human Genetics, David Geffen School of Medicine, UCLA, Los Angeles, CA, USA.

This PDF file includes: Figures S1–S11, Table S1, Supplementary Methods for Fig. S11, and References for the SI Appendix.

Supplemental Figures and Figure Legends

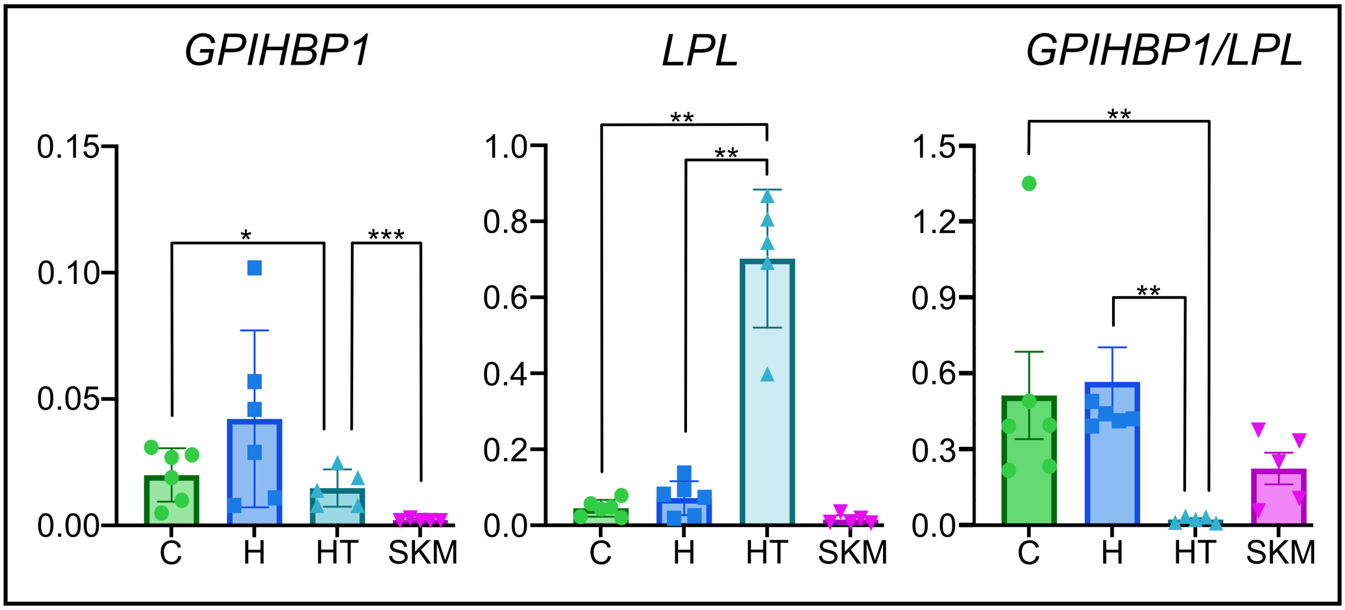

Fig. S1. Quantification by RT-PCR of *GPIHBP1* and *LPL* transcripts (normalized to GAPDH) in human cerebral cortex (C; n = 6), hippocampus (H; n = 6), heart (HT; n = 5), and skeletal muscle (SKM; n = 5). Statistical significance was assessed by one-way ANOVA with Tukey's multiple-comparisons test. Mean ± S.D. *$P < 0.05$; **$P < 0.01$; ***$P < 0.001$.

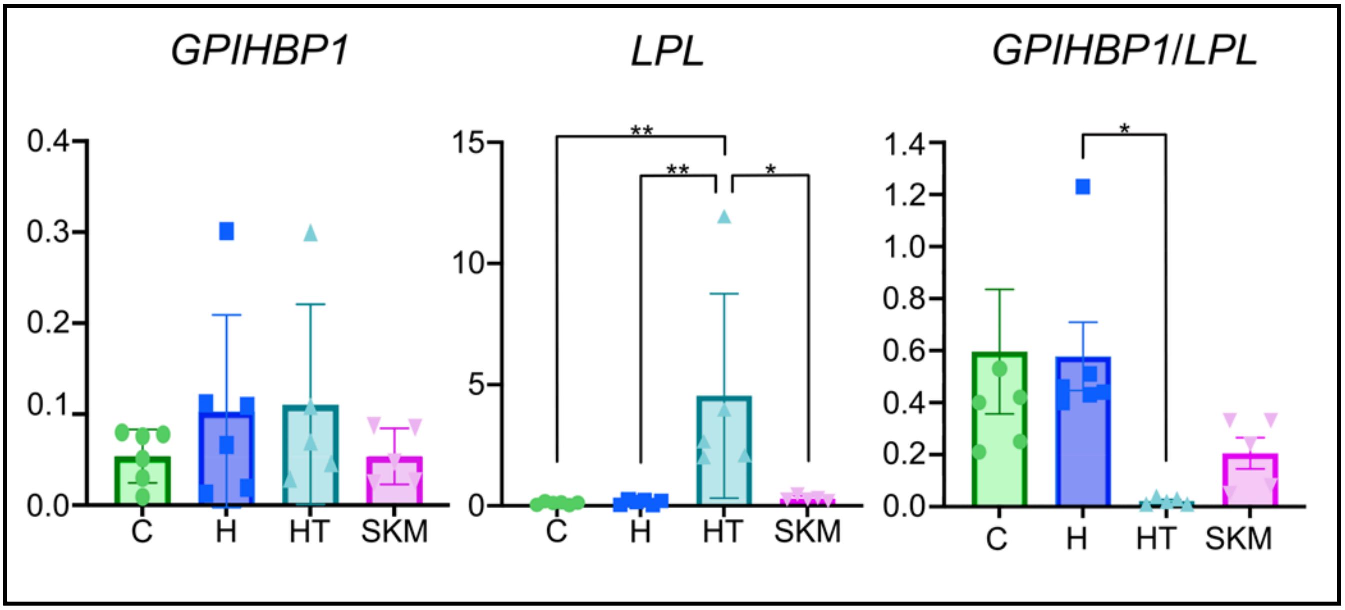

Fig. S2. Quantification by RT-PCR of *GPIHBP1* and *LPL* transcripts (normalized to *TUBB*) in human cerebral cortex (C; n = 6), hippocampus (H; n = 6), heart (HT; n = 5), and skeletal muscle (SKM; n = 5). Statistical significance was assessed by one-way ANOVA with Tukey's multiple-comparisons test. Mean ± S.D. *P < 0.05; **P < 0.01.

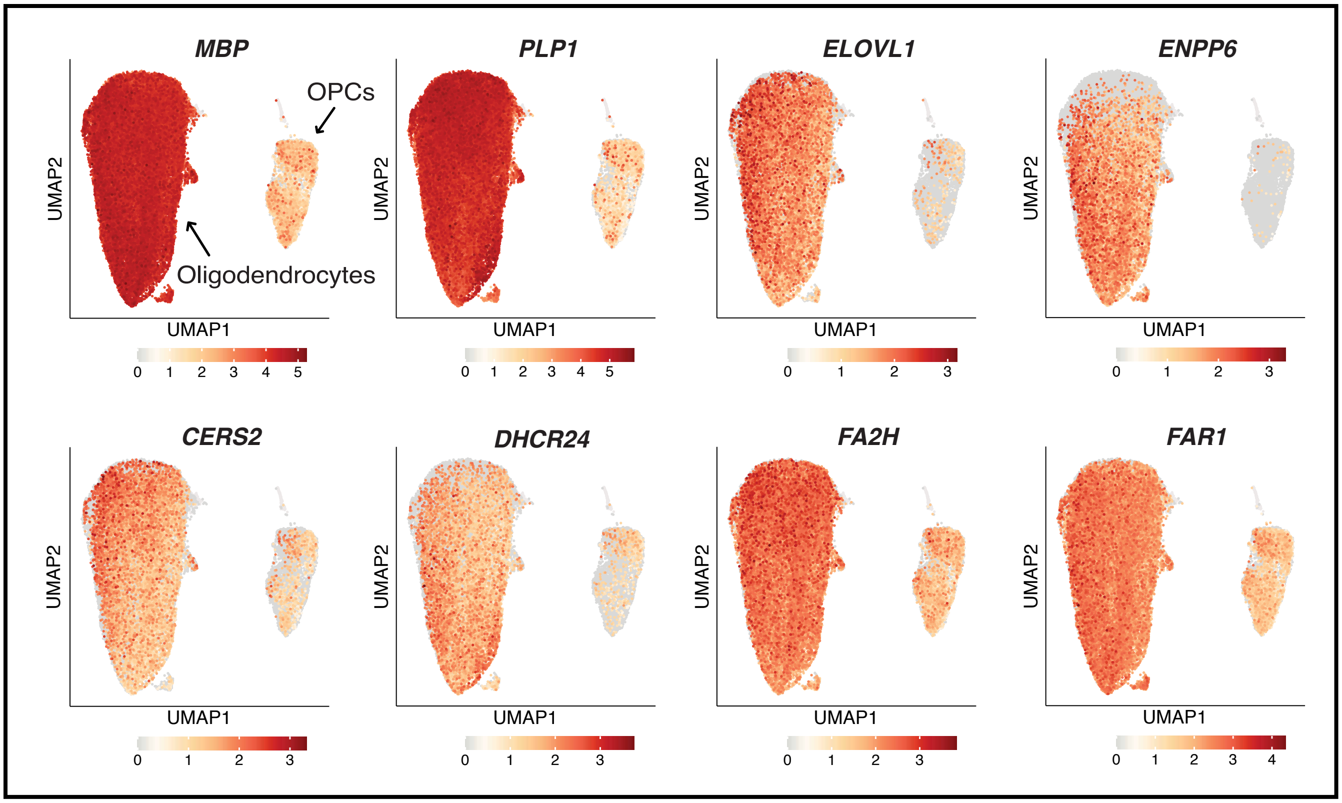

Fig. S3. Expression of genes linked to hypomyelination or demyelination in oligodendrocytes and oligodendrocyte precursor cells (OPCs). UMAP plots of human brain single-nuclei RNA-seq data from Siletti et al. (1) (https://github.com/linnarsson-lab/adult-human-brain; file: human_adult-GRCh38-3.0.0.h5ad) illustrating the expression patterns of genes associated with hypomyelination or demyelination (*MBP, PLP1, ELOV1, ENPP6, CERS2, DHCR24, FA2H, FAR1*) (2-12). 56,614 cells were analyzed.

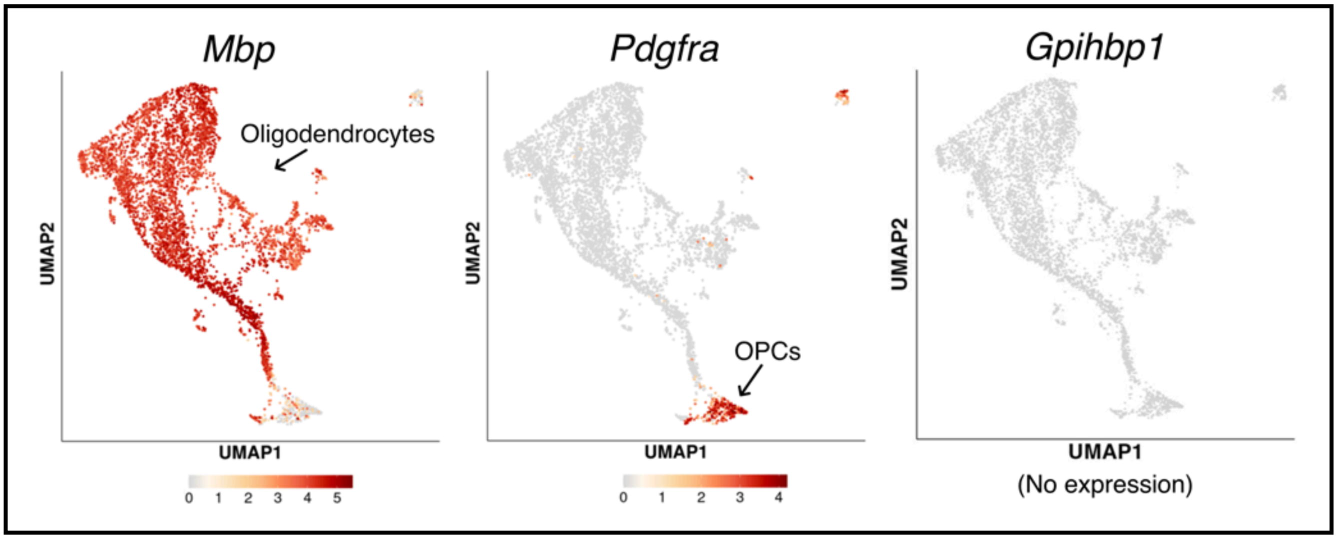

Fig. S4. Absent expression of *Gpihbp1* in mouse oligodendrocytes. UMAP plots of single-cell RNA-seq data from juvenile and adult mouse brain (GSE75330; 5,069 cells analyzed) (13) showing expression of *Mbp* (oligodendrocyte marker), *Pdgfra* (OPC marker), and *Gpihbp1*. In the middle panel, the small group of *Pdgfra*-positive cells in the upper right-hand corner are vascular and leptomeningeal cells distinct from OPCs.

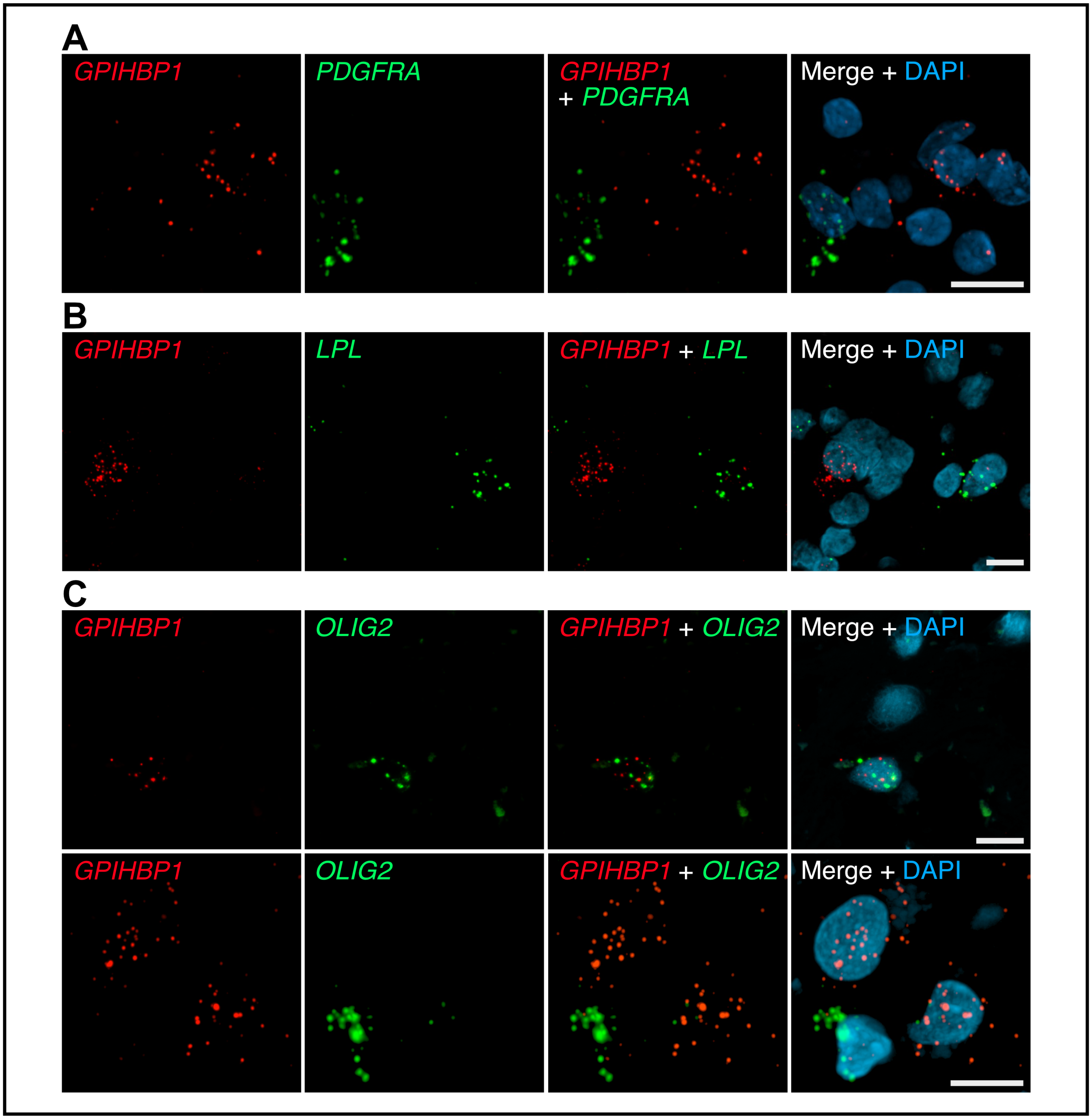

Fig. S5. *In situ* hybridization studies of *GPIHBP1*, *PDGFRA*, *LPL*, and *OLIG2* expression in human hippocampus. (**A**) *GPIHBP1* transcripts (Dye 570; *red*) and *PDGFRA* transcripts (Dye 650; *green*); (**B**) *GPIHBP1* transcripts (Dye 570; *red*) and *LPL* transcripts (Dye 650; *green*); and (**C**) *GPIHBP1* transcripts (Dye 570; *red*) and *OLIG2* transcripts (Dye 650; *green*). For all sample, nuclei were stained with Dapi (*blue*). RNAscope probes were from ACD Bio-Techne. Scale bars, 10 µm.

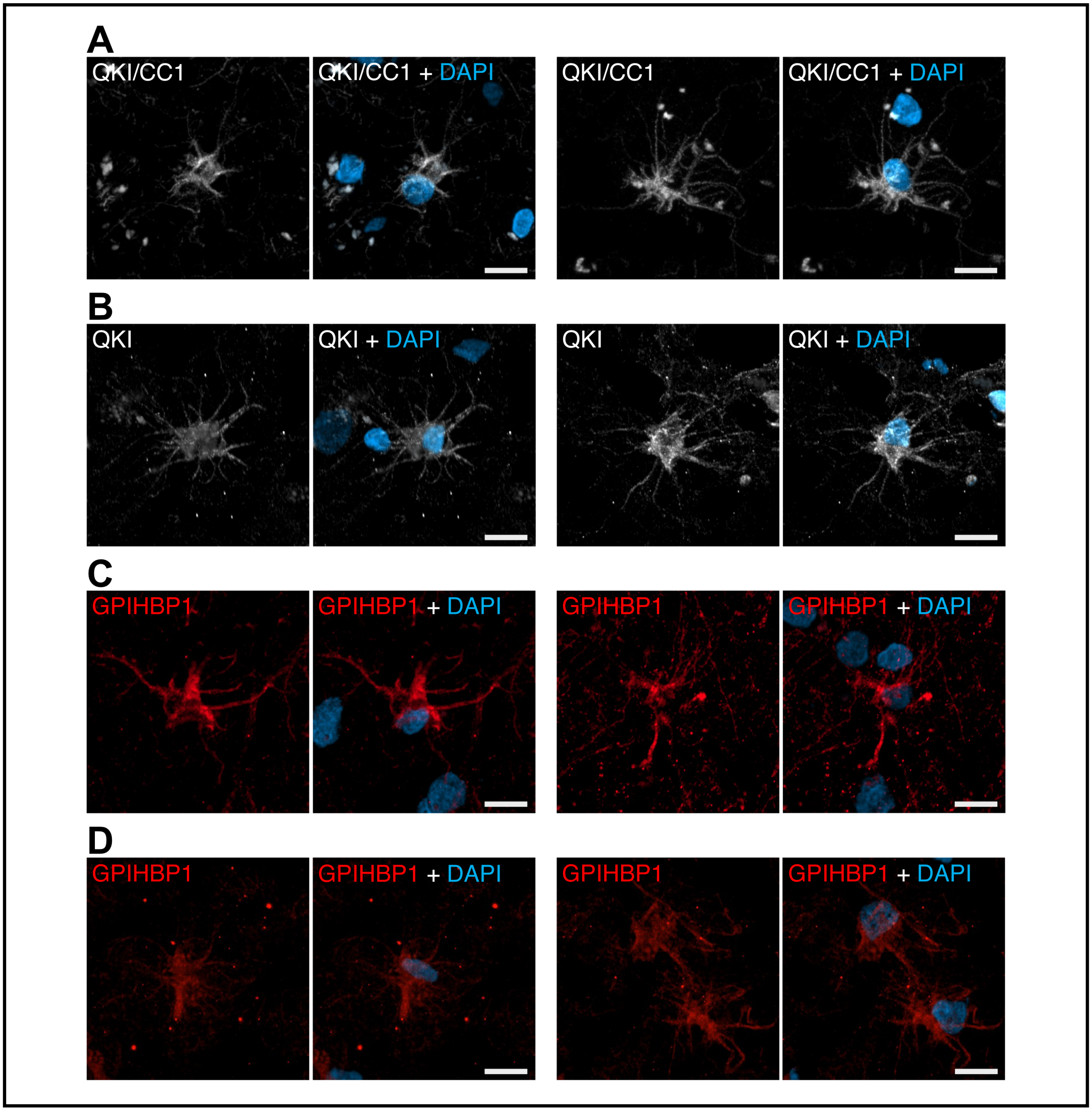

Fig. S6. Immunofluorescence staining of human hippocampus with two independent antibodies for QKI and GPIHBP1. (**A**) QKI was detected with a mouse mAb against QKI (CC1) (14) followed by an Alexa Fluor 568–labeled donkey antibody against mouse IgG (*white*). (**B**) QKI expression was detected with a mouse mAb against QKI (Abcam, ab186245) followed by an Alexa Fluor 568–labeled donkey antibody against mouse IgG (*white*). (**C**) GPIHBP1 was detected with a polyclonal rabbit antibody against GPIHBP1 followed by an Alexa Fluor 647–labeled donkey antibody against rabbit IgG (*red*). (**D**) GPIHBP1 was detected with a mixture of three different mouse mAbs against human GPIHBP1(RF4, RE3, RG3) followed by an Alexa Fluor 568–labeled donkey antibody against mouse IgG (*red*). For all samples, nuclei were stained with Dapi (*blue*). Scale bars, 10 µm.

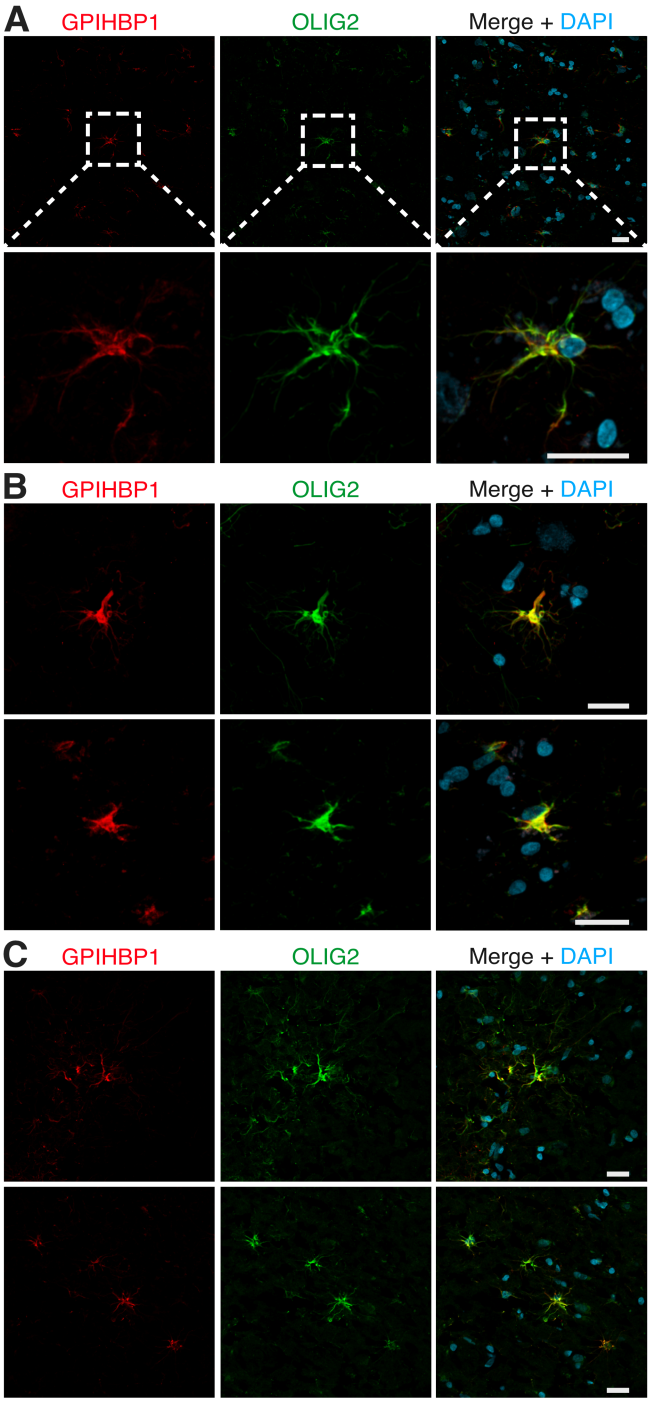

Fig. S7. Colocalization of GPIHBP1 and OLIG2 in different human brain regions. Immunofluorescence staining of human frontal cortex (**A**), corpus callosum (**B**), and hippocampus (**C**). GPIHBP1 was detected with a human GPIHBP1-specific rabbit IgG followed by an Alexa Fluor 568–labeled donkey antibody against rabbit IgG (*red*). OLIG2 was detected with an OLIG2-specific mouse mAb followed by an Alexa Fluor 647–labeled donkey antibody against mouse IgG (*green*). Nuclei were stained with Dapi (*blue*). Two fields are shown for the corpus callosum and hippocampus. OLIG2 is a nuclear protein, but it escapes into the cytoplasm after death (15). Scale bars, 20 μm.

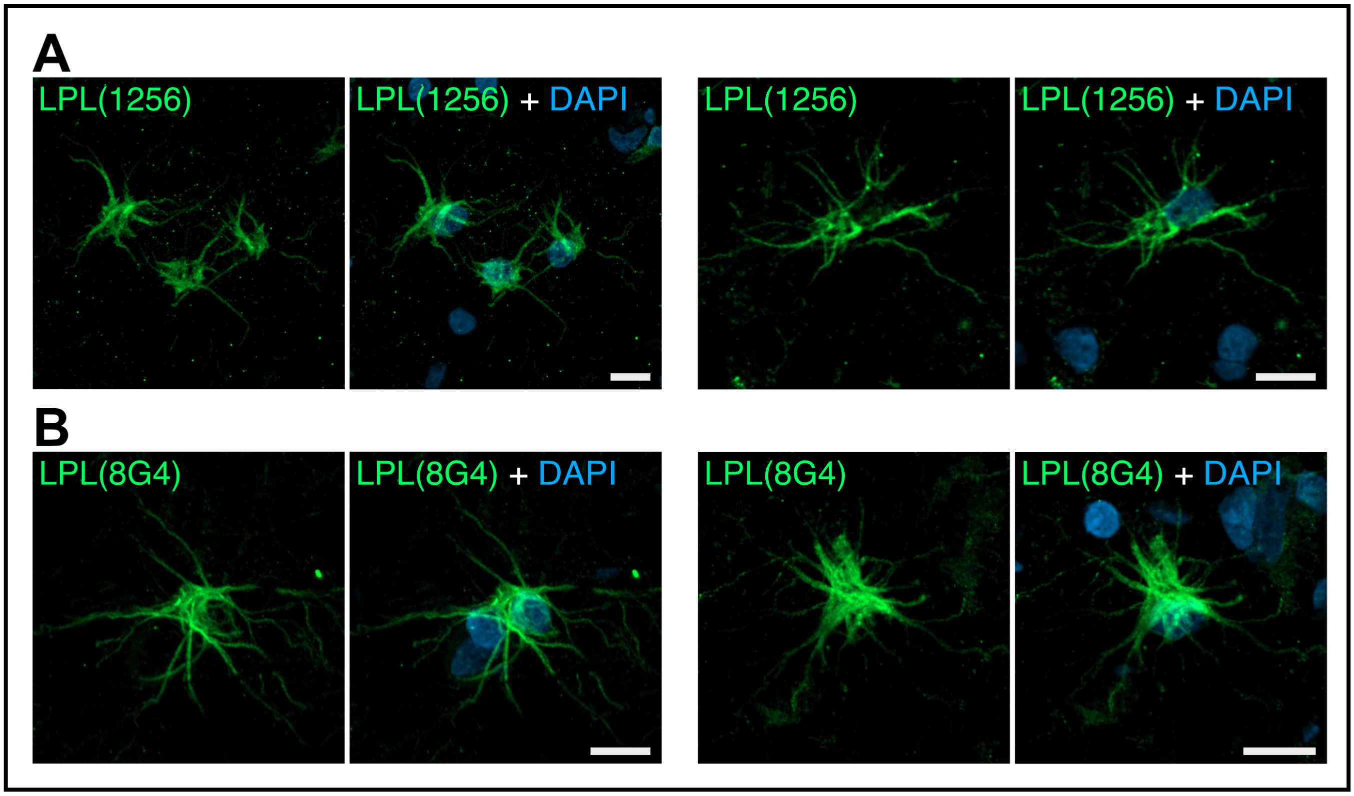

Fig. S8. Immunofluorescence staining of human hippocampus with two LPL-specific antibodies. (**A**) LPL detected with a polyclonal rabbit antibody against human LPL (1256) followed by an Alexa Fluor 488–labeled donkey antibody against rabbit IgG (*green*). Two fields are shown. (**B**) LPL detected with a mouse mAb against human LPL (8G4) followed by an Alexa Fluor 488–labeled donkey antibody against mouse IgG (*green*). Nuclei are stained with Dapi (*blue*). Two fields are shown. Scale bars, 10 µm.

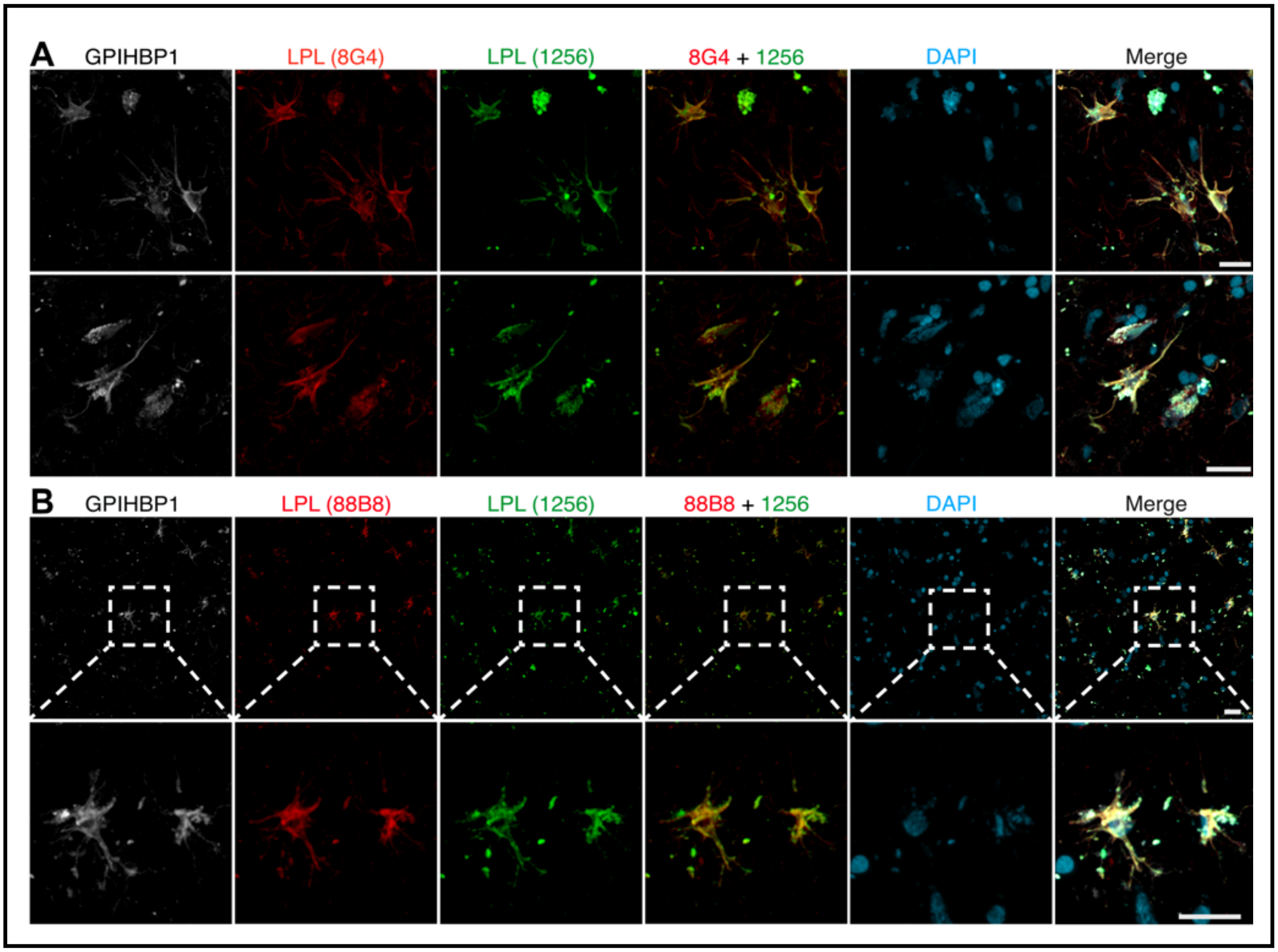

Fig. S9. Immunohistochemistry studies demonstrating colocalization of GPIHBP1 and LPL (detected with different LPL-specific antibodies) in human hippocampus. GPIHBP1 was detected with a rabbit polyclonal IgG against human GPIHBP1 followed by an Alexa Fluor 568–labeled donkey antibody against rabbit IgG (*white*). LPL was detected with Alexa Fluor 488–labeled polyclonal rabbit antibody against human LPL (1256; *green*). LPL was also detected with human LPL–specific mouse mAbs (Panel A: 8G4; Panel B: 88B8) followed by an Alexa Fluor 568–labeled donkey antibody against mouse IgG (*red*). Nuclei are stained with Dapi (*blue*). Scale bars, 20 μm.

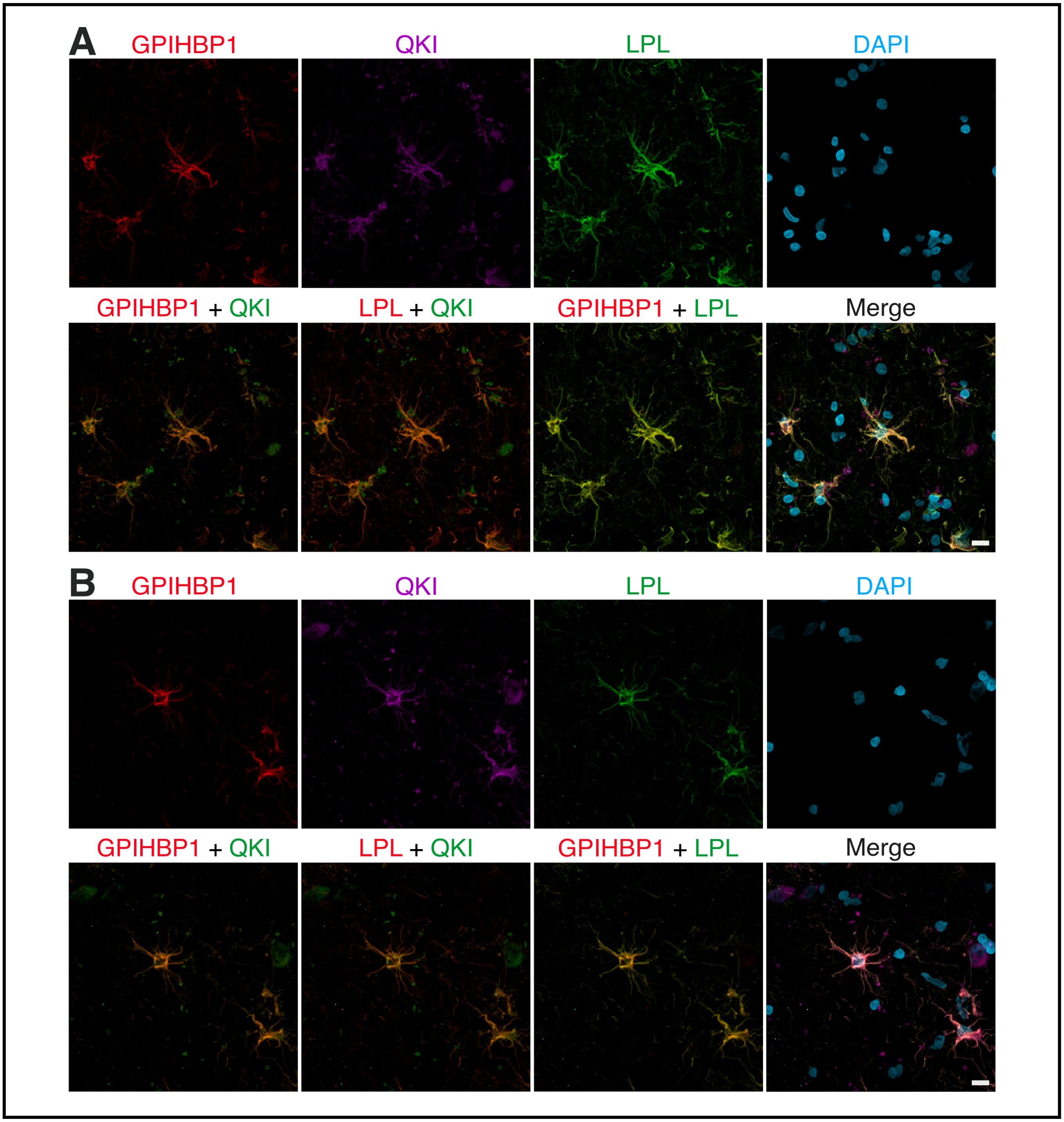

Fig. S10. Immunofluorescence staining of human hippocampus demonstrating colocalization of GPIHBP1, LPL, and QKI. QKI was detected with a mouse mAb against QKI (CC1) followed by an Alexa Fluor 568–labeled donkey antibody against mouse IgG (*white*). LPL was detected with a rabbit antibody against human LPL (1256) followed by an Alexa Fluor 488–labeled donkey antibody against rabbit IgG (*green*). GPIHBP1 was detected with an Alexa Fluor 647–labeled rabbit IgG against human GPIHBP1 (*red*). Nuclei were stained with Dapi (*blue*). Scale bars, 10 µm.

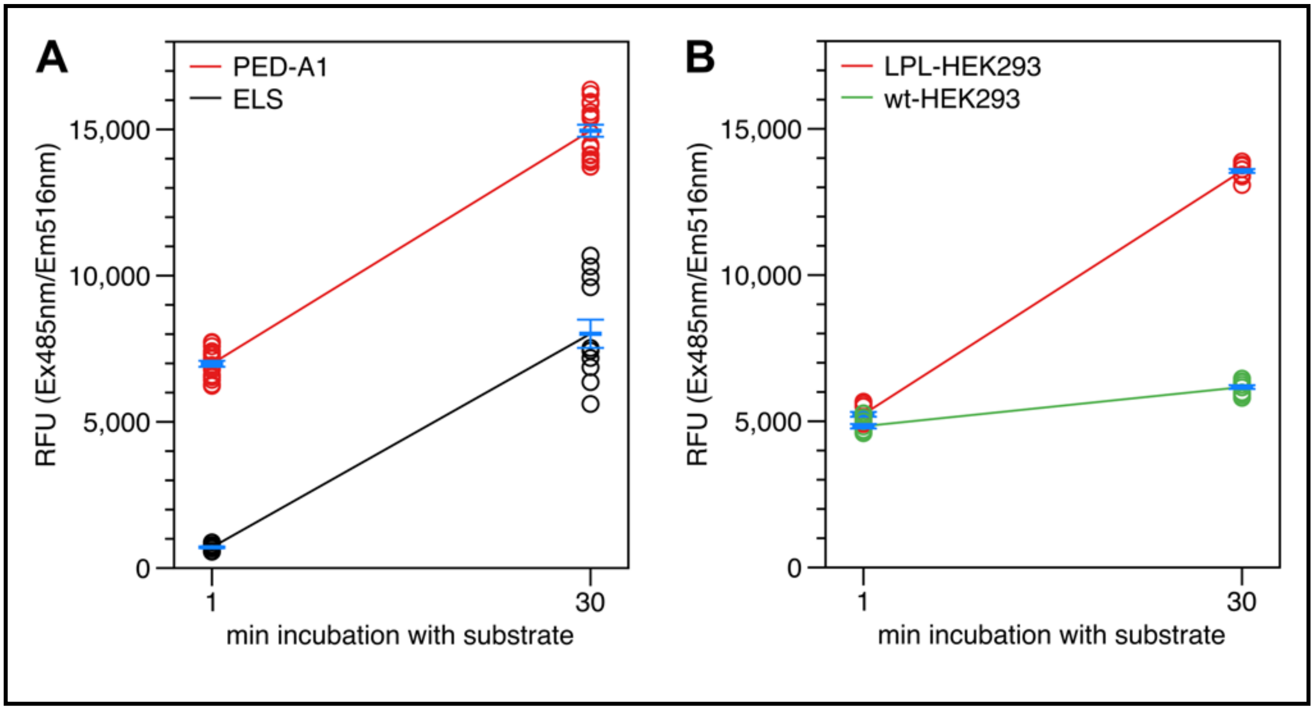

Fig. S11. A cell-based assay of human LPL activity with the EnzChek BODIPY-labeled TG analog ELS and the BODIPY-labeled EnzChek phospholipase A1–selective substrate PED-A1. (**A**) Testing the ability of the human LPL to hydrolyze ELS and PED-A1 substrates. HEK293 cells stably expressing human LPL were seeded at a density of 50,000 cells/well into 96-well plates (Costar). After an overnight incubation, the medium was replaced with 80 μl of medium (for the ELS-based assay) or 80 μl of OptiMEM serum-free medium (for the PED-A1–based assay). Cells were incubated for 60 min before adding 20 μl of 5× ELS substrate solution or 20 μl of 5× PED-A1 substrate solution. Fluorescence was assessed at 1 min and 30 min with a Synergy Neo2 plate reader with an excitation wavelength of 485 nm and an emission wavelength of 516 nm. Plotted are the fluorescence values 1 min and 30 min after adding the ELS substrate (n = 14) or the PED-A1 substrate (n = 20). Blue bars show mean ± SEM. (**B**) Testing the capacity of medium from non-transfected HEK293 cells (wt-HEK293) and the human LPL-expressing HEK293 cells (LPL-HEK293) to hydrolyze the phospholipase A1–selective substrate PED-A1. This experiment was performed according to the procedures outlined for panel A. Florescence was assessed in 48 independent wells. After a 30-min incubation of LPL-HEK293 cells in the medium with the PED-A1 substrate, fluorescence increased sharply, indicating LPL phospholipase activity. In contrast, we observed minimal increase in fluorescence with the medium from wt-HEK293 cells.

Methods for the LPL Activity Assays in Fig. S11

A TG hydrolysis assay (16) and a phospholipase assay (17) were performed as previously described. Cell-based assays were used to assess both LPL's TG hydrolase activity and its phospholipase activity. The EnzChek lipase substrate (ELS, a BODIPY-dabcyl-labeled TG analog) (ThermoFisher) was used to assess TG hydrolysis; phospholipase activity was assessed with the phospholipase A1-selective substrate PED-A1 [N-((6-(2,4-DNP)amino)hexanoyl)-1-BODIPYTM-FL-C5)-2-hexyl-*sn*-glycero-3-phosphoethanolamine] (ThermoFisher). HEK 293 cells stably expressing human LPL were maintained in growth medium [DMEM/F12 (3:1) (Invitrogen), 10% FBS (Hyclone)] and seeded at a density of 50,000 cells/well in 96-well plates (Costar). After an overnight incubation at 37°C, the medium was replaced with 80 µl of growth medium for the ELS-based assay and 80 µl of OptiMEM serum-free medium for the PED-A1–based assay. Cells were incubated for 60 min before adding 20 µl of either 5× ELS or 5× PED-A1 working solutions. The 5× ELS working solution was prepared with ELS substrate and 0.05% Zwittergent detergent 3-(N,N-dimethyl-octadecylammonio)-propanesulfonate (Sigma) and added to the wells to achieve a final concentration of 1 µM. The 5× PED-A1 working solution consisting of the PED-A1 substrate, DOPC (1,2-dioleoyl-*sn*-glycero-3-phosphocholine), and DOPG [1,2-dioleoyl-*sn*-glycero-3-phospho-rac-(1-glycerol) sodium salt] (Sigma) and added to the cells to achieve concentrations of 1, 10, and 10 µM, respectively. Fluorescence was monitored at 1 and 30 minutes with a Synergy Neo2 plate reader with an excitation wavelength of 485 nm and an emission wavelength of 516 nm.

Table S1. RT-PCR primers.

**Target**	**Forward (5′→3′)**	**Reverse (5′→3′)**
GPIHBP1	AGACCTGCACAACCCTCATT	CTCCACCGTCTTGGTGATG
LPL	TAGCTGGTCAGACTGGTGGA	TTCACAAATACCGCAGGTG
GAPDH	GCTCTCTGCTCCTCCTGTTC	ACGACCAAATCCGTTGACTC

References for the *SI Appendix*

1. K. Siletti *et al.*, Transcriptomic diversity of cell types across the adult human brain. *Science* **382**, eadd7046 (2023).
2. H. Bagheri *et al.*, Myelin basic protein mRNA levels affect myelin sheath dimensions, architecture, plasticity, and density of resident glial cells. *Glia* **72**, 1893-1914 (2024).
3. G. Khalaf *et al.*, Mutation of Proteolipid Protein 1 Gene: from severe hypomyelinating leukodystrophy to inherited spastic paraplegia. *Biomedicines* **10** (2022).
4. M. Isokawa, T. Sassa, S. Hattori, T. Miyakawa, A. Kihara, Reduced chain length in myelin sphingolipids and poorer motor coordination in mice deficient in the fatty acid elongase Elovl1. *FASEB Bioadv* **1**, 747-759 (2019).
5. K. M. Wong *et al.*, Biallelic ELOVL1 variants are linked to hypomyelinating leukodystrophy, movement disorder, and ichthyosis. *Mov Disord* **40**, 1836-1850 (2025).
6. J. Morita *et al.*, Structure and biological function of ENPP6, a choline-specific glycerophosphodiester-phosphodiesterase. *Sci Rep* **6**, 20995 (2016).
7. S. Imgrund *et al.*, Adult ceramide synthase 2 (CERS2)-deficient mice exhibit myelin sheath defects, cerebellar degeneration, and hepatocarcinomas. *J Biol Chem* **284**, 33549-33560 (2009).
8. J. D. Teo *et al.*, Early microglial response, myelin deterioration and lethality in mice deficient for very long chain ceramide synthesis in oligodendrocytes. *Glia* **71**, 1120-1141 (2023).
9. S. Edvardson *et al.*, Mutations in the fatty acid 2-hydroxylase gene are associated with leukodystrophy with spastic paraparesis and dystonia. *Am J Hum Genet* **83**, 643-648 (2008).
10. L. Cao, X. J. Huang, C. J. Chen, S. D. Chen, A rare family with Hereditary Spastic Paraplegia Type 35 due to novel FA2H mutations: a case report with literature review. *J Neurol Sci* **329**, 1-5 (2013).
11. T. Takahashi, K. Otsuka, T. Sassa, A. Kihara, Acyl-CoA reductase Far1 deficiency impairs ether lipid production and hypomyelination in mouse brains. *Mol Cell Biol* **45**, 495-510 (2025).
12. J. Zolotushko *et al.*, The desmosterolosis phenotype: spasticity, microcephaly and micrognathia with agenesis of corpus callosum and loss of white matter. *Eur J Hum Genet* **19**, 942-946 (2011).
13. S. Marques *et al.*, Oligodendrocyte heterogeneity in the mouse juvenile and adult central nervous system. *Science* **352**, 1326-1329 (2016).
14. J. M. Bin, S. N. Harris, T. E. Kennedy, The oligodendrocyte-specific antibody 'CC1' binds Quaking 7. *J Neurochem* **139**, 181-186 (2016).
15. F. Parisi, S. Degl'Innocenti, C. Aytas, A. Pirone, C. Cantile, Morphological and immunohistochemical changes in progressive postmortem autolysis of the murine brain. *Animals (Basel)* **14** (2024).
16. Y. Q. Chen *et al.*, Angiopoietin-like protein 8 differentially regulates ANGPTL3 and ANGPTL4 during postprandial partitioning of fatty acids. *J Lipid Res* **61**, 1203-1220 (2020).
17. Y. Q. Chen *et al.*, Angiopoietin-like protein 4 (ANGPTL4) is an inhibitor of endothelial lipase (EL) while the ANGPTL4/8 complex has reduced EL-inhibitory activity. *Heliyon* **7**, e07898 (2021).
